# Local Phase Transitions in a Model of Multiplex Networks with Heterogeneous Degrees and Inter-Layer Coupling

**DOI:** 10.3390/e25050828

**Published:** 2023-05-22

**Authors:** Nedim Bayrakdar, Valerio Gemmetto, Diego Garlaschelli

**Affiliations:** 1 Lorentz Institute for Theoretical Physics, University of Leiden, 2333 CA Leiden, The Netherlands; 2 IMT School of Advanced Studies Lucca, 55100 Lucca, Italy; 3 INdAM-GNAMPA Istituto Nazionale di Alta Matematica, 00185 Rome, Italy

**Keywords:** multiplex networks, maximum entropy models, World Trade Multiplex, mean-field Ising model

## Abstract

Multilayer networks represent multiple types of connections between the same set of nodes. Clearly, a multilayer description of a system adds value only if the multiplex does not merely consist of independent layers. In real-world multiplexes, it is expected that the observed inter-layer overlap may result partly from spurious correlations arising from the heterogeneity of nodes, and partly from true inter-layer dependencies. It is therefore important to consider rigorous ways to disentangle these two effects. In this paper, we introduce an unbiased maximum entropy model of multiplexes with controllable intra-layer node degrees and controllable inter-layer overlap. The model can be mapped to a generalized Ising model, where the combination of node heterogeneity and inter-layer coupling leads to the possibility of local phase transitions. In particular, we find that node heterogeneity favors the splitting of critical points characterizing different pairs of nodes, leading to link-specific phase transitions that may, in turn, increase the overlap. By quantifying how the overlap can be increased by increasing either the intra-layer node heterogeneity (spurious correlation) or the strength of the inter-layer coupling (true correlation), the model allows us to disentangle the two effects. As an application, we show that the empirical overlap observed in the International Trade Multiplex genuinely requires a nonzero inter-layer coupling in its modeling, as it is not merely a spurious result of the correlation between node degrees across different layers.

## 1. Introduction

The wide variety of different phenomena that occur around us are often the result of systems that emerge and (self-)organize dynamically. These systems consist of a multitude of basic constituents interacting with each other in complicated ways and forming complex patterns. Many of these systems can be represented as networks sustaining various processes. Examples of such systems include social networks, transportation networks, biological networks, financial networks, and technological networks. In particular, social, financial, and economic networks are an important class of systems that, in the wake of recent global crises (such as the 2007–2008 financial crisis, the COVID-19 pandemic, and the ongoing Ukraine crisis), have been attracting attention given the possibility of studying the propagation of shocks among their constituents. Generally, individuals, banks, firms, or countries can be represented as nodes, and the relationships among them can be represented as links [1,2,3]. Other types of economic and financial networks are obtained as some form of projection from time series data [3,4,5,6,7]. The study of these networks may increase our understanding of a variety of processes that take place through them, such as the spreading of diseases, the diffusion of (mis)information, the stability of financial markets, and the resilience of the economy.

The simplest approach is to map each constituent within a system onto a single node and to map each interaction between pairs of constituents onto a link of a single type, regardless of the nature of the interaction. In this approach, all the links in a network are treated on an equal footing, making it a single-layer network representation, which might, however, lead to an oversimplification that fails to capture the details of a multirelational system. For instance, production and trade networks are the result of the functioning of global supply chains, involving the exchange of multiple products between firms and countries, which determines nontrivial dependencies between product-specific layers of the network. In order to realistically follow the propagation of shocks in the economy, knowledge of the nature of the links is essential. The inability to properly represent multirelational systems using single-layer networks has lead to the introduction of so-called *multilayer networks* [8,9,10,11,12]. Multilayer networks allow us to describe multirelational systems by representing each type of relationship in a separate layer of the network, where each node is present in all layers, and the different types of connections are reported in the corresponding layers. Returning to the example of social networks, the different types of relationships between people, such as kinship, friendship, coworkership, etc., would each be represented by links in a different layer [13], and could be analyzed in their mutual dependencies.

However, in order to assess true dependencies across layers, one should use proper null models. In recent years, there has been an increase in attention towards null models of networks constructed as random graph ensembles [14,15,16,17,18,19,20]. A class of such models is the so-called Exponential Random Graph Models (ERGMs) [17,18,19,20,21,22,23,24,25,26,27]. ERGMs are used commonly within the social network analysis community, and have been more recently re-derived within a statistical physics maximum entropy framework [19,20,27]. This has allowed researchers to utilize techniques that are common in statistical physics. In the ERGM framework, one chooses the probability distribution on graphs such that it maximizes the entropy. This maximization is performed while the expected values of certain chosen graph properties are constrained to be equal to desired values.

Real-world multilayer networks have been compared against null ERGMs with independent layers [28,29]. This comparison has highlighted various properties of real multilayer networks that result from the interdependence of layers. Two such properties are the *overlap* and the *multiplexity* [9,28]. The overlap and the multiplexity essentially contain similar information and capture the correlation of a node’s connectivity across two or more layers. For example, in a social network, people may communicate with their friends through multiple means of communication, such as talking on the phone, sending emails, or sending instant text messages. In this example, the layer that represents communication through email has a significant overlap with the layer of communication through text messages. A more specific example is a study of the so-called World Trade Multiplex (representing international trade in different commodities among countries [30]), which showed that, despite the fact that each layer of the multiplex is separately well described by a maximum entropy model with given node degrees [31,32,33], the observed trade overlap across different commodity-specific layers is significantly different from the overlap predicted by a null model with independent layers [28]. This result is not unexpected, since one can imagine that the trade of a certain product between two countries may increase/decrease the possibility of the trade of a *different* product between the same two countries. Other examples of networks displaying a significant overlap are airport networks, on-line social games, collaboration networks, and citation networks [34,35,36].

An important conclusion that has been reached after comparing real-world multiplexes against null models with independent layers is that a significant part of the observed overlap in many real networks could actually be spuriously created by the correlations among node degrees across different layers, even if the latter are conditionally independent of each other, instead of resulting from genuine inter-layer dependencies [28,29]. Indeed, if node degrees are correlated among layers, then there will be an increased probability of a link between two nodes being present in multiple layers, while the probability of a link occurring in one layer will not necessarily influence the presence of a link occurring in another layer. The measured overlap of the network therefore consists of a part resulting from ‘spurious’ coupling between the layers and of a part resulting from genuine coupling between the layers. This spurious coupling increases as the density and/or heterogeneity of the degrees of the network increases. Real-world networks are often dense and have strongly heterogeneous degrees; therefore, the assessment of inter-layer coupling in these real-world networks will be severely affected.

The focus of this paper is the introduction of interdependencies between the layers of a multilayer network in the ERGM through the explicit inclusion of the overlap as an extra constraint. This inclusion of the overlap in the ERGM will aid us in understanding which (higher-order) properties of the network structure may be (highly) dependent on the overlap. Additionally, it will help us distinguish between the overlap in the network due to the correlation of single-node properties across layers and the overlap due to a genuine coupling between the layers. Finally, it will allow us to generate null models with the desired amount of spurious overlap and genuine overlap. It turns out that this problem is mathematically identical to solving the Ising model on a complete graph (which is also known as the mean-field Curie–Weiss model) and leads to a phase transition between a ‘multiplexed’ (magnetized) and a ‘non-multiplexed’ (non-magnetized) phase. However, the problem is more general because the locality of the constraints on the degrees of nodes will imply different parameter values, and hence different properties for the phase transitions relative to different pairs of nodes. For instance, it will, in general, not be possible to enforce a ‘zero-field’ spontaneous symmetry breaking condition for all pairs of nodes simultaneously. Therefore, for a given specification of the constraints, different pairs of nodes may realize different symmetry-broken values of their contribution to the overall inter-layer overlap. Crucially, this property arises only from the simultaneous presence of the two constraints (on the global overlap and on the heterogeneous local degrees), and would not be realized in the absence of one of them.

The rest of the paper is organized as follows: In Section 2, we mathematically define quantities and models that are relevant to this paper. This includes the derivation of a benchmark model, where the layers of the multiplex network are independent. In Section 3, we introduce, and solve analytically, our new model, where the layers of the multiplex are interdependent due to the inclusion of the overlap. Section 4 contains a discussion regarding the possible local phase transitions of the model. In Section 5, we explore our model by using various numerical methods. In Section 6, we briefly analyze the World Trade Multiplex, and show that the empirical overlap in this real-world network is not merely the result of the heterogeneity of the network, but requires a nonzero coupling between the layers in its modeling. Finally, we provide some concluding remarks in Section 7, and some technical details in Appendix A and Appendix B.

## 2. Background Theory

This section contains some background notions, definitions, and models.

### 2.1. Single-Layer Network Definitions

We will limit our discussion to the case of *binary and undirected* networks. A binary undirected network can be defined as a graph that is an ordered pair G=(V,E), where $V=\{v_{1},v_{2},...,v_{N}\}$ is a set of *N vertices* or *nodes*, and *E* is a set of *unordered* pairs of different vertices called *edges* or *links*. Note that the definition of *E* depends on the relevant class of relations between the constituents of the system. The vertex $v_{i}\in V$ will be referred to simply as *i* throughout the rest of the paper. If (i,j)∈E, the vertices *i* and *j* are said to be connected, and may be referred to as *neighbors* of each other. The number of links *L* of the graph is given by the cardinality of *E*: L=|E|.

Matrix Representation

A graph *G* is represented by its *adjacency matrix* $G=\{g_{ij}\}$. This is an N×N matrix where

(1) $$g_{ij}=\left\{\begin{array}{cc} 1 & \mathrm{if}\;(i,j)\in E,\\ 0 & \mathrm{otherwise}. \end{array}\right.$$

We define *E* as containing pairs of *distinct* vertices, which means that a vertex cannot have a connection to itself (self-loop). It is then natural to define the diagonal elements as $g_{ii}\equiv 0$. Since we limit our discussion to undirected graphs, the adjacency matrix is always symmetric, $g_{ij}=g_{ji}$, and it therefore contains N(N−1)/2 independent elements that fully specify the matrix and ultimately the graph.

Degrees and Degree Distribution

One of the main topics in the analysis of complex networks is the identification of the different roles that nodes play [37]. For instance, there are a variety of measures that characterize the structural importance of a node in a network. The degree $k_{i}\left(G\right)$ of the graph *G* is defined as the number of connections node *i* has to other nodes in the network.

(2) $$k_{i}\left(G\right)=\sum_{j=1}^{N}g_{ij}$$

The list ${\left\{k_{i}\left(G\right)\right\}}_{i=1}^{N}$ of degrees is called the *degree sequence* of the graph *G*. The degree distribution P(k) is defined as the fraction of nodes in the network with degree *k*. Real-world networks systematically show a degree distribution with heavy tails, where the degrees vary over a broad range, often spanning several orders of magnitude [38,39]. The majority of the vertices of these real-world networks have a small number of links to other vertices, while a few vertices have a relatively high number of links to other vertices, which are also referred to as ‘hubs’. An example is the World Wide Web, where some pages are incredibly popular and are pointed to by thousands of other pages, while generally, most pages are almost unknown. The heavy tails of real-world degree distributions can often be, but not necessarily, approximated by power laws of the form $P\left(k\right)∼k^{-\gamma }$. In any case, vertices with a degree much larger than the average degree 〈k〉 occur with a non-negligible probability. This is a signature of a high level of statistical heterogeneity in real-world networks. Encoding this heterogeneity will be a crucial ingredient of our models.

### 2.2. Multiplex Network Definitions

A binary undirected multiplex network can be defined in terms of the previously defined single-layer networks. A multiplex network is a set $\vec{G}={\left\{G^{\alpha }\right\}}_{\alpha =1}^{M}$ of *M* undirected binary graphs $G^{\alpha }=\left(V,E^{\alpha }\right)$ that share the same set of *N* nodes. In the context of multilayer networks, $G^{\alpha }$ is called a layer of M, and will be referred to simply as α throughout the rest of the paper. Note that a multiplex network is a type of multilayer network that does not allow inter-layer connections between two layers α and β where α≠β.

Matrix Representation

The layer $G^{\alpha }$ and its intra-layer links can then be represented by the adjacency matrix $G^{\alpha }=\left\{g_{ij}^{\alpha }\right\}$. This is an N×N matrix where

(3) $$g_{ij}^{\alpha }=\left\{\begin{array}{cc} 1 & \mathrm{if}\;\left(i,j\right)\in E^{\alpha },\\ 0 & \mathrm{otherwise}. \end{array}\right.$$

Multilinks in Multiplex Networks

In order to capture the information regarding the presence of the links between the pair of nodes (i,j) in any of the *M* layers, we define the object

(4) $$m_{ij}\equiv \left(g_{ij}^{1},\;g_{ij}^{2},\;\ldots ,\;g_{ij}^{M}\right)$$

which is also known as the *multilink* of (i,j). Additionally, we define the set $M_{ij}$ as the set that contains all the $2^{M}$ possible configurations of $m_{ij}$.

Multidegrees

The multidegree of a node i∈V of a multiplex network $\vec{G}$ is the object

(5) $${\vec{k}}_{i}\left(\vec{G}\right)\equiv \left(k_{i}^{1}\left(\vec{G}\right),\;k_{i}^{2}\left(\vec{G}\right),\;\ldots ,\;k_{i}^{M}\left(\vec{G}\right)\right)$$

where

(6) $$k_{i}^{\alpha }\left(\vec{G}\right)=\sum_{j\neq i}^{N}g_{ij}^{\alpha }$$

is the degree of the node *i* in the layer α [9,40]. From the vector definition of the multidegree, one can obtain a scalar quantity defined as the *layer-averaged degree*:

(7) $${\bar{k}}_{i}\left(\vec{G}\right)=\frac{1}{M}\sum_{\alpha =1}^{M}k_{i}^{\alpha }\left(\vec{G}\right),$$

which is the degree of node *i* averaged over all the *M* layers. Note that, in each layer α, the total layer-specific degree of all nodes equals twice the number of links in that layer, which we denote as $L^{\alpha }$:

(8) $$\sum_{i=1}^{N}k_{i}^{\alpha }\left(\vec{G}\right)=\sum_{i<j}g_{ij}^{\alpha }=2L^{\alpha }\left(\vec{G}\right).$$

Summing the above relationship for the *M* layers, we get

(9) $$M\sum_{i=1}^{N}{\bar{k}}_{i}\left(\vec{G}\right)=\sum_{\alpha =1}^{M}\sum_{i<j}g_{ij}^{\alpha }=2\sum_{\alpha =1}^{M}L^{\alpha }\left(\vec{G}\right)=2L\left(\vec{G}\right),$$

where $L(\vec{G})$ denotes the total number of links over the entire multiplex:

(10) $$L\left(\vec{G}\right)=\sum_{\alpha =1}^{M}\sum_{i<j}g_{ij}^{\alpha }.$$

Overlap

There are many properties that encode the interdependence between the layers of a multilayer network, but we will limit our discussion to one such property: the overlap. The overlap $O^{\alpha \beta }\left(\vec{G}\right)$ between two layers α and β of the multiplex $\vec{G}$ is defined as the number of links that appear in both layers α and β [34,41]:

(11) $$O^{\alpha \beta }\left(\vec{G}\right)=\sum_{i<j}g_{ij}^{\alpha }g_{ij}^{\beta }$$

where, throughout the paper, using $\sum_{a<b}$ and $\prod_{a<b}$, we denote a *double* sum and a *double* product for all possible (unrepeated) pairs of values of the two indices, *a* and *b* (with a≠b), respectively. The *global overlap* $O(\vec{G})$ is defined as the sum of $O^{\alpha \beta }\left(\vec{G}\right)$ for all pairs of layers:

(12) $$O\left(\vec{G}\right)=\sum_{\alpha <\beta }\sum_{i<j}g_{ij}^{\alpha }g_{ij}^{\beta }.$$

As the names of these properties suggest, they are a measure of how overlapping the layers of the multiplex network are.

### 2.3. Exponential Random Graph Models for Multiplexes

ERGMs are ensemble models, which means that they are defined as probability distributions over many possible (multiplex) networks. Given the observed (or desired) value $C_{i}^{*}\equiv C_{i}\left(\vec{G}*\right)$ for *K* graph properties ${\left\{C_{i}\left(\vec{G}\right)\right\}}_{i=1}^{K}$ defined on each possible multiplex $\vec{G}$ (where $\vec{G}*$ represents a particular, e.g., real-world, multiplex of interest), an ERGM generates a probability distribution $P(\vec{G})$ over multiplex networks that maximizes the entropy, under the constraint that the expected value of $C_{i}\left(\vec{G}\right)$ equals $C_{i}^{*}$, for all i=1,K. This method provides us with a general framework for modeling maximally random (maximum entropy) multiplex networks, to be used as null models that can be compared against the empirical multiplex $\vec{G}*$ to detect higher-order patterns that are irreducible to the *K* enforced constraints. Maximizing the entropy subject to a set of constraints is also widely used in problems with incomplete information [42,43].

Let $G_{N}^{M}$ be the set of (binary undirected) multiplex networks consisting of *N* vertices and *M* layers (note that this set includes single-layer networks for M=1), let $\vec{G}=\left\{G_{1},G_{2},...,G_{M}\right\}\in G_{N}^{M}$ be a multiplex network in that set, and let $P(\vec{G})$ be the sought-for probability of $\vec{G}$ within the ensemble. We want $P(\vec{G})$ to be such that the expectation value of each graph observable $C_{i}\left(\vec{G}\right)$ (in the chosen set of *K* observables) is equal to the corresponding observed or desired value $C_{i}^{*}$. This type of probability distribution is also referred to as a *canonical ensemble*. The ideal probability distribution is the one that maximizes the Gibbs–Shannon entropy

(13) $$S=-\sum_{\vec{G}\in G_{N}^{M}}P\left(\vec{G}\right)\ln P(\vec{G})$$

under the normalization condition

(14) $$\sum_{\vec{G}\in G_{N}^{M}}P\left(\vec{G}\right)=1$$

and the other *K* constraints

(15) $$C_{i}^{*}=\langle C_{i}\rangle ,\;i=1,\ldots ,K,$$

where

(16) $$\langle C_{i}\rangle \equiv \sum_{\vec{G}\in G_{N}^{M}}P\left(\vec{G}\right)C_{i}\left(\vec{G}\right).$$

The maximization of the entropy is achieved by introducing a global Lagrange multiplier η for the normalization condition and a specific multiplier $\theta_{i}$ for each constraint $\langle C_{i}\rangle =C_{i}^{*}$, i=1,…,K. This leads to the parametric solution

(17) $$P\left(\vec{G},\vec{\theta }\right)=\frac{e^{-H(\vec{G},\vec{\theta })}}{Z(\vec{\theta })}$$

where $H(\vec{G},\vec{\theta })$ is the graph Hamiltonian

(18) $$H\left(\vec{G},\vec{\theta }\right)\equiv \sum_{i=1}^{K}\theta_{i}C_{i}\left(\vec{G}\right)=\vec{\theta }\cdot \vec{C}\left(\vec{G}\right)$$

and $Z(\vec{\theta })$ is the partition function determined by the normalization condition

(19) $$Z\left(\vec{\theta }\right)\equiv e^{\eta +1}=\sum_{\vec{G}\in G_{N}^{M}}e^{-H(\vec{G},\vec{\theta })}.$$

The parametric form of $P(\vec{G},\vec{\theta })$, if inserted back into Equation (13), leads to the explicit expression for the entropy:

(20) $$S\left(\vec{\theta }\right)=-\sum_{\vec{G}\in G_{N}^{M}}P\left(\vec{G},\vec{\theta }\right)\ln P(\vec{G},\vec{\theta })=\vec{\theta }\cdot \langle \vec{C}\rangle +\ln Z\left(\vec{\theta }\right).$$

### 2.4. Maximum Likelihood Parameter Estimation

Equations (17)–(19) fully define the ERGM, apart from the specification of the parameters $\vec{\theta }$. In principle, by treating these Lagrange multipliers as free parameters, one can study the effects that the specification of certain graph observables $\left\{C_{i}\right\}$ has on other aspects of network structure [27,44,45,46,47]. This approach, however, does not allow one to consider ERGMs as null models of a particular real network [17,19]. In the latter case, maximum likelihood parameter estimation leads to the unique (given the choice of constraints) ERGM representing a null model for a particular real (multiplex) network $\vec{G}*$, and hence, enforcing Equation (15) exactly, as we briefly recall below. This null model can then be used to detect statistically significant deviations of empirical structural properties of $\vec{G}*$ from the ensemble.

The log-likelihood of the particular multiplex $\vec{G}*$ is

(21) $$L\left(\vec{G}*,\vec{\theta }\right)=\ln P(\vec{G}*,\vec{\theta })=-\sum_{i=1}^{K}\theta_{i}C_{i}^{*}-\ln Z(\vec{\theta }).$$

This function has the following properties [19]:

(22) $$\frac{\partial L(\vec{G}*,\vec{\theta })}{\partial \theta_{i}}=\langle C_{i}\rangle -C_{i}^{*}$$

(23) $$\begin{array}{cc} \frac{\partial^{2}L\left(\vec{G}*,\vec{\theta }\right)}{\partial \theta_{i}\;\partial \theta_{j}} & =-\langle C_{i}C_{j}\rangle +\langle C_{i}\rangle \langle C_{j}\rangle . \end{array}$$

Equation (22) means that the stationary points $\vec{\theta }=\vec{\theta }*$ of L are precisely those that satisfy the constraints (15), i.e.,

(24) $${\langle C_{i}\rangle }_{\vec{\theta }*}=\sum_{\vec{G}\in G_{N}^{M}}C_{i}\left(\vec{G}\right)P\left(\vec{G},\vec{\theta }*\right)=\sum_{\vec{G}\in G_{N}^{M}}C_{i}\left(\vec{G}\right)\frac{e^{-\sum_{j=1}^{K}\theta_{j}^{*}C_{j}\left(\vec{G}\right)}}{Z(\vec{\theta })}=C_{i}\left(\vec{G}*\right),\;i=1,\ldots ,K$$

where ${\langle C_{i}\rangle }_{\vec{\theta }*}$ indicates that the ensemble average is evaluated at the values $\vec{\theta }*$. Equation (23) indicates that L is concave, since the matrix with entries $\partial^{2}L/\partial \theta_{i}\;\partial \theta_{j}$ has the form of a negative covariance matrix, and must therefore be non-positive definite [48]. The solutions $\vec{\theta }*$ of the coupled equations ${\langle C_{i}\rangle }_{\vec{\theta }*}=C_{i}^{*}$ in Equation (15) can therefore be found by maximizing the log-likelihood L. If $\partial^{2}L/\partial \theta_{i}\;\partial \theta_{j}$ is negative definite, which will be true if the functions $C_{i}\left(\vec{G}\right)$ are linearly independent [48] (i.e., the chosen constraints are non-redundant), then there will be, at most, one solution, and it will be the unique maximum of L. Maximizing a concave function is generally easier than solving the system of coupled nonlinear equations in Equation (24). Once the solution $\vec{\theta }=\vec{\theta }*$ is found, it can be used to generate a null model of $\vec{G}*$. Moreover, inserting the value $\theta^{*}$ back into Equation (21) and using Equation (20), we obtain the important relation

(25) $$\begin{array}{ccc} L(\vec{G}*,\vec{\theta }*) & = & \ln P(\vec{G}*,\vec{\theta }*)\\ & = & -\sum_{i=1}^{K}\theta_{i}^{*}C_{i}^{*}-\ln Z(\vec{\theta }*)\\ & = & -\sum_{i=1}^{K}\theta_{i}^{*}{\langle C_{i}\rangle }_{\vec{\theta }*}-\ln Z(\vec{\theta }*)\\ & = & -S(\vec{\theta }*), \end{array}$$

i.e., the maximized log-likelihood equals minus the entropy for the particular value $\vec{\theta }*$, which in turn represents the ‘entropy of the data’ given the chosen constraints. This result allows one to easily calculate the entropy of the data $S\left(\vec{\theta }*\right)=-L\left(\vec{G}*,\vec{\theta }*\right)$ automatically as part of the likelihood maximization procedure, rather than as a much more complicated formal sum of all configurations, as in the general definition (13).

### 2.5. Benchmark: Independent Layers Model

As anticipated in the Introduction, our goal is that of considering how the empirical overlap between links in different layers of a multiplex is jointly determined by both a ‘genuine’ coupling between the *M* layers and a ‘spurious’ correlation resulting from the heterogeneous (and correlated across layers) degrees of the *N* nodes. As a null benchmark before inserting both components in an ERGM of a multiplex, we first consider only the layer-averaged degrees of all vertices as constraints, as defined in Equation (7). We can therefore create a null model of a real multiplex $\vec{G}*$ using the ERGM in combination with the maximum likelihood method. This model will be referred to as the *Average Configuration Model (ACM)*, and will allow us to study the sole effects of correlated heterogeneous degrees on the inter-layer overlap. The Hamiltonian of this model, denoted as $H_{0}$, since it represents a benchmark for a more complicated model to be defined later, is

(26) $$H_{0}\left(\vec{G},\vec{\theta }\right)=M\sum_{i=1}^{N}\theta_{i}{\bar{k}}_{i}\left(\vec{G}\right)=\sum_{\alpha =1}^{M}\sum_{i<j}\left(\theta_{i}+\theta_{j}\right)g_{ij}^{\alpha }$$

where we have reparametrized by exposing *M* for convenience. The partition function is

(27) $$\begin{array}{cc} Z_{0}\left(\vec{\theta }\right) & =\sum_{\vec{G}\in G_{N}^{M}}e^{-\sum_{\alpha =1}^{M}\sum_{i<j}\left(\theta_{i}+\theta_{j}\right)g_{ij}^{\alpha }}\\ & =\sum_{\vec{G}\in G_{N}^{M}}\prod_{\alpha =1}^{M}\prod_{i<j}e^{-\left(\theta_{i}+\theta_{j}\right)g_{ij}^{\alpha }}\\ & =\prod_{\alpha =1}^{M}\prod_{i<j}\sum_{g_{ij}^{\alpha }=0}^{1}e^{-\left(\theta_{i}+\theta_{j}\right)g_{ij}^{\alpha }}\\ & =\prod_{\alpha =1}^{M}\prod_{i<j}\left[1+e^{-(\theta_{i}+\theta_{j})}\right]\\ & =\prod_{i<j}{\left[1+e^{-(\theta_{i}+\theta_{j})}\right]}^{M}. \end{array}$$

The probability distribution over the ensemble is then given by

(28) $$P_{0}\left(\vec{G},\vec{\theta }\right)=\prod_{\alpha =1}^{M}\prod_{i<j}\frac{e^{-\left(\theta_{i}+\theta_{j}\right)g_{ij}^{\alpha }}}{1+e^{-(\theta_{i}+\theta_{j})}},$$

from which we see that pairs of nodes and pairs of layers are all independent of each other, each entry $g_{ij}^{\alpha }$ being an independent Bernoulli random variable with success probability $p_{ij}^{\alpha }\left(\vec{\theta }\right)$ and expected value ${\langle g_{ij}^{\alpha }\rangle }_{\vec{\theta }}$ given by

(29) $$p_{ij}^{\alpha }\left(\vec{\theta }\right)={\langle g_{ij}^{\alpha }\rangle }_{\vec{\theta }}=\frac{e^{-(\theta_{i}+\theta_{j})}}{1+e^{-(\theta_{i}+\theta_{j})}}\equiv p_{ij}\left(\vec{\theta }\right).$$

Clearly, $p_{ij}^{\alpha }\left(\vec{\theta }\right)=p_{ij}\left(\vec{\theta }\right)$ is the probability that a link occurs between node *i* and *j* in layer α, which turns out to be independent of α given our choice of the layer-averaged (not layer-specific) degree as a constraint.

The log-likelihood of the multiplex $\vec{G}*$ is

(30) $$L_{0}\left(\vec{G}*,\vec{\theta }\right)=-M\sum_{i=1}^{N}\theta_{i}{\bar{k}}_{i}^{*}-M\sum_{i<j}\ln\left[1+e^{-(\theta_{i}+\theta_{j})}\right],$$

where ${\bar{k}}_{i}^{*}={\bar{k}}_{i}\left({\vec{G}}^{*}\right)$. The parameter value $\theta_{m}^{*}$ maximizing the log-likelihood must satisfy

(31) $$\begin{array}{cc} {\left.\frac{\partial L_{0}\left(\vec{G}*,\vec{\theta }\right)}{\partial \theta_{m}}\right|}_{\vec{\theta }=\vec{\theta }*}\; & =-M{\bar{k}}_{m}^{*}+M\sum_{j\neq m}\frac{e^{-(\theta_{m}^{*}+\theta_{j}^{*})}}{1+e^{-(\theta_{m}^{*}+\theta_{j}^{*})}}=0\;\forall m \end{array}$$

or equivalently,

(32) $${\bar{k}}_{i}^{*}=\sum_{j\neq i}\frac{e^{-(\theta_{i}^{*}+\theta_{j}^{*})}}{1+e^{-(\theta_{i}^{*}+\theta_{j}^{*})}}\;\forall i.$$

The above results show that, as expected from the general result reported in Equation (24), according to the maximum likelihood principle, the empirical layer-averaged degree ${\bar{k}}_{i}^{*}={\bar{k}}_{i}\left(\vec{G}*\right)$ of the real multiplex $\vec{G}*$ is equal to the ensemble average ${\langle {\bar{k}}_{i}\rangle }_{\vec{\theta }*}$:

(33) $$\begin{array}{cc} {\bar{k}}_{i}^{*} & =\sum_{j\neq i}p_{ij}\left(\vec{\theta }*\right)\\ & =\frac{1}{M}\sum_{\alpha =1}^{M}\sum_{j\neq i}p_{ij}^{\alpha }\left(\vec{\theta }*\right)\\ & =\frac{1}{M}\sum_{\alpha =1}^{M}\sum_{j\neq i}{\langle g_{ij}^{\alpha }\rangle }_{\vec{\theta }*}\\ & ={\langle {\bar{k}}_{i}\rangle }_{\vec{\theta }*}. \end{array}$$

The probability distribution $P_{0}\left(\vec{G},\vec{\theta }*\right)$ can then be written as a product of the layers:

(34) $$P_{0}\left(\vec{G},\vec{\theta }*\right)=\prod_{\alpha =1}^{M}P_{0}^{\alpha }\left(G^{\alpha },\vec{\theta }*\right)$$

where $P_{0}^{\alpha }$ is the probability distribution over a single layer, i.e.,

(35) $$P_{0}^{\alpha }\left(G^{\alpha },\vec{\theta }*\right)=\prod_{i<j}{\left[p_{ij}\left(\vec{\theta }*\right)\right]}^{g_{ij}^{\alpha }}{\left[1-p_{ij}\left(\vec{\theta }*\right)\right]}^{1-g_{ij}^{\alpha }}.\;$$

This means that each layer α can be generated by using the link probability $p_{ij}\left(\vec{\theta }*\right)$ that is equal throughout the layers. This is again a consequence of exclusively constraining properties defined as the overall averages of the layers. This null model can be used as a benchmark to determine the expected value of the inter-layer overlap $O(\vec{G})$ defined in Equation (12), which is due solely to the correlation between the degree of the same node *i* across the *M* layers, and not to any genuine inter-layer dependency. This expected value is

(36) $${\langle O\rangle }_{\vec{\theta }*}=\sum_{\alpha <\beta }\sum_{i<j}{\langle g_{ij}^{\alpha }g_{ij}^{\beta }\rangle }_{\vec{\theta }*}=\sum_{\alpha <\beta }\sum_{i<j}{\langle g_{ij}^{\alpha }\rangle }_{\vec{\theta }*}{\langle g_{ij}^{\beta }\rangle }_{\vec{\theta }*}=\sum_{\alpha <\beta }\sum_{i<j}p_{ij}^{2}\left(\vec{\theta }*\right),\;$$

where we have used the independence ${\langle g_{ij}^{\alpha }g_{ij}^{\beta }\rangle }_{\vec{\theta }*}={\langle g_{ij}^{\alpha }\rangle }_{\vec{\theta }*}\langle g_{ij}^{\beta }\rangle$ between layers α≠β. Deliberately, we have chosen the layer-averaged degree as the only constraint so that the expected degree of a node is the same across all layers, thereby creating a strong correlation between degrees in different layers, while keeping the layers themselves independent. Using Equations (25) and (30), we can calculate the entropy of the data, given the model, as

(37) $$S_{0}\left(\vec{\theta }*\right)=-L_{0}\left(\vec{\theta }*\right)=-\ln P_{0}\left(\vec{G}*,\vec{\theta }*\right)=M\sum_{i=1}^{N}\theta_{i}^{*}{\bar{k}}_{i}^{*}+M\sum_{i<j}\ln\left[1+e^{-(\theta_{i}^{*}+\theta_{j}^{*})}\right],$$

which only requires the knowledge of $\vec{\theta }*$ and of the layer-averaged degrees ${\bar{k}}_{i}\left(\vec{G}*\right)$, i=1,N.

## 3. The Overlapping Average Configuration Model

Having illustrated all the ingredients that are necessary to define and model basic properties of multiplex networks within a maximum entropy framework, in this section, we introduce a model of multiplex networks with genuinely interdependent layers. To this end, we incorporate the overlap as an extra constraint in the ERGM, and study the model in combination with the maximum likelihood method. This model is a generalization of the previous ACM benchmark, and will therefore be referred to as the *Overlapping Average Configuration Model (OACM)*, as it includes not only the intra-layer degrees, but also the inter-layer coupling, as building blocks.

### 3.1. Constructing the Hamiltonian

We want to define a model of a multiplex with *M* layers, *N* vertices, and given expected layer-averaged degrees (as defined in Equation (7)) and global inter-layer overlap (as defined in Equation (12)). The Hamiltonian of our ERGM is, in this case,

(38) $$H\left(\vec{G},\vec{\theta },J\right)=M\sum_{i=1}^{N}\theta_{i}{\bar{k}}_{i}\left(\vec{G}\right)-\frac{4J}{M}O\left(\vec{G}\right)=\sum_{i<j}\sum_{\alpha =1}^{M}\left(\theta_{i}+\theta_{j}\right)g_{ij}^{\alpha }-\frac{4J}{M}\sum_{i<j}\sum_{\alpha <\beta }g_{ij}^{\alpha }g_{ij}^{\beta }\;$$

where $\left(\vec{\theta },J\right)$ are the Lagrange multipliers coupled to the N+1 constraints. We have defined the Lagrange multiplier for the overlap as −4J/M for later convenience. Clearly, $H\left(\vec{G},\vec{\theta },J\right)=H_{0}\left(\vec{G},\vec{\theta }\right)$ where $H_{0}$ is the benchmark Hamiltonian of the ACM without overlap defined in Equation (26). Using the multilink $m_{ij}$ defined in Equation (4) and defining

(39) $$\theta_{ij}\equiv \theta_{i}+\theta_{j},$$

the Hamiltonian in Equation (38), this can be written as a sum of the pairs of vertices:

(40) $$H\left(\vec{G},\vec{\theta },J\right)=\sum_{i<j}h_{ij}\left(m_{ij},\theta_{ij},J\right)$$

where

(41) $$h_{ij}\left(m_{ij},\theta_{ij},J\right)\equiv \left(\theta_{i}+\theta_{j}\right)\sum_{\alpha =1}^{M}g_{ij}^{\alpha }-\frac{4J}{M}\sum_{\alpha <\beta }g_{ij}^{\alpha }g_{ij}^{\beta }$$

will be referred to as the *pair Hamiltonian*. As we shall see in a moment, the pair Hamiltonian can be mapped exactly to a mean-field Ising model coupling the *M* layers homogeneously. To arrive at this mapping, we transform the Boolean variables $g_{ij}^{\alpha }\in \left\{0,1\right\}$ to new ‘spin’ variables $\sigma_{ij}^{\alpha }\in \left\{-1,1\right\}$, as follows:

(42) $$g_{ij}^{\alpha }=\frac{1}{2}\left(\sigma_{ij}^{\alpha }+1\right).$$

From now on, we assume that *M* is large (multiplex with several layers) and expand expressions accordingly. By defining

(43) $$s_{ij}\equiv \left\{\sigma_{ij}^{1},\sigma_{ij}^{2},\ldots ,\sigma_{ij}^{M}\right\}$$

as the multilink for the node pair (i,j) in terms of the $\sigma_{ij}^{\alpha }=\pm 1$ variables, we see that Equation (42) can be used to transform Equation (41) into

(44) $$h_{ij}\left(s_{ij},\theta_{ij},J\right)=\left(\frac{\theta_{ij}}{2}-J\right)\sum_{\alpha =1}^{M}\sigma_{ij}^{\alpha }-\frac{J}{M}\sum_{\alpha <\beta }\sigma_{ij}^{\alpha }\sigma_{ij}^{\beta }-\frac{JM}{2}+\frac{M\theta_{ij}}{2}.$$

If we define

(45) $$B_{ij}\equiv J-\frac{\theta_{ij}}{2},$$

(46) $$v_{ij}\equiv -MB_{ij}+\frac{JM}{2},$$

then the pair Hamiltonian finally reduces to

(47) $$h_{ij}\left(s_{ij},B_{ij},J\right)=-B_{ij}\sum_{\alpha =1}^{M}\sigma_{ij}^{\alpha }-\frac{J}{M}\sum_{\alpha <\beta }\sigma_{ij}^{\alpha }\sigma_{ij}^{\beta }+v_{ij}.$$

From the above expression, we see that, for every specific pair of nodes (i,j), the variables $\sigma_{ij}^{\alpha }$ can be thought of as Ising spins residing in the *M* nodes of a fully connected graph, where every Ising spin interacts with every other M−1 spins and is coupled to a ‘field’ $B_{ij}$. In terms of the multiplex networks being modeled, this means that for every specific pair of nodes (i,j), the edges connecting *i* and *j* throughout the *M* layers are all coupled to a common ‘external’ field $B_{ij}$, and are also coupled to each other with a homogeneous interaction strength J/M. A positive coupling J>0 favors more overlap (i.e., more alignment between links in different layers), while J<0 disfavors the overlap. The term $v_{ij}$ is an inessential overall shift in energy independent of the spin configuration. This model is identical to the mean-field Ising or Curie–Weiss model. This exact mapping is what we use in Appendix in order to solve the model analytically, and in particular, to show the existence, for each pair of nodes, of a phase transition separating a ‘magnetized’ phase and a ‘non-magnetized’ phase, which here represent a ‘multiplexed’ phase (where links in different layers tend to ‘align’ to each other) and a ‘non-multiplexed’ phase, respectively.

The full Hamiltonian (40) is a summation of the Hamiltonians of *non-interacting* Ising systems, each for a distinct pairs of nodes. Note, however, that despite the independence of different pairs of nodes, the pair Hamiltonians $h_{ij}\left(s_{ij},B_{ij},J\right)$ share some parameters: *J* is common to all such Hamiltonians, and $h_{ij}\left(s_{ij},B_{ij},J\right)$ and (say) $h_{ik}\left(s_{ik},B_{ik},J\right)$ also share the parameter $\theta_{i}$, because the latter appears in both $B_{ij}$ and $B_{ik}$. This is the result of the original constraint on the degree of each node, which results in the same Lagrange multiplier $\theta_{i}$ appearing in all pair Hamiltonians involving the same node *i*. These common parameters imply that, even if all pairs of nodes are independent, the control parameters of all pair Hamiltonians cannot be chosen independently, resulting in a correlated phenomenology for the various pairs of nodes. In particular, as we shall see, each pair of nodes can undergo *locally* the typical phase transition of the mean-field Ising model, but the features of these pair-specific phase transitions are all nontrivially related to each other.

We also note, from Equations (44) and (47), that if $J=\theta_{ij}/2$ (or equivalently, $B_{ij}=0$), then the pair Hamiltonian (hence the graph probability) becomes invariant upon a global ‘spin flip’ ($\sigma_{ij}^{\alpha }\to -\sigma_{ij}^{\alpha }$ ∀α), which here corresponds to the replacement of each existing link with a missing link ($g_{ij}^{\alpha }=1\to g_{ij}^{\alpha }=0$ ∀α) and, vice versa, of each missing link with an existing link ($g_{ij}^{\alpha }=0\to g_{ij}^{\alpha }=1$ ∀α). This is due to the vanishing of the ‘external field’ $B_{ij}$ that, when present, selects a preferred ‘spin direction’ (up versus down), which here means a preferred density (high versus low). We expect that with the parameter choice $J=\theta_{ij}/2$, the pair of nodes (i,j) gains an expected 1/2 density of links across the *M* layers, i.e., an expected number of links equal to M/2, corresponding to half the maximum number of links for that node pair. Additionally, if *J* is smaller than the critical value, this expected number of links is also the typical value, and basically, the model is not fundamentally different from a model without constraints, where the intermediate density is produced as a result of a completely uniform probability distribution for the multilink. However, if *J* exceeds the critical value, the intermediate average density is no longer the typical one realized by individual graphs sampled from the model: rather, it is the ensemble average of two typical (high and low) values of the realized density, just like in the equivalent spin system, below the Curie temperature, and without an external field one would typically observe, with the same probability, overall positive and negative magnetization with a zero ensemble average. The numerical simulations access the typical realized values, while the equations still govern the expected value. This situation corresponds to a ‘symmetry-broken’ phase, where the typical realizations are less symmetric than the Hamiltonian that generates them. However, here, the heterogeneity of the degrees implies different values of the external field $B_{ij}=J-\theta_{ij}/2$, which means that the zero-field spontaneous symmetry breaking condition cannot, in general, be realized for all pairs of nodes simultaneously, leading to a phenomenology governed by the interplay between the values of *J* and ${\left\{\theta_{i}\right\}}_{i=1}^{N}$, and ultimately between the values of the inter-layer overlap and the node degrees.

### 3.2. Calculating the Partition Function

The partition function defined in (19) can be written as the product

(48) $$Z\left(\vec{\theta },J\right)=\sum_{\vec{G}\in G_{N}^{M}}e^{-H(\vec{G},\vec{\theta },J)}=\sum_{\vec{G}\in G_{N}^{M}}\prod_{i<j}e^{-h_{ij}\left(s_{ij},\theta_{ij},J\right)}=\prod_{i<j}z_{ij}\left(\theta_{ij},J\right),$$

where $z_{ij}\left(\theta_{ij},J\right)$ is the *pair partition function*, which is a sum of the set $S_{ij}$ of all $2^{M}$ possible multilinks for (i,j):

(49) $$z_{ij}\left(\theta_{ij},J\right)\equiv \sum_{s_{ij}\in S_{ij}}e^{-h_{ij}\left(s_{ij},\theta_{ij},J\right)}.$$

The multiplex probability can be written in terms of the multilink probabilities $P_{ij}\left(s_{ij},\theta_{ij},J\right)$:

(50) $$P(\vec{G},\vec{\theta },J)=\prod_{i<j}P_{ij}\left(s_{ij},\theta_{ij},J\right)$$

where

(51) $$P_{ij}\left(s_{ij},\theta_{ij},J\right)\equiv \frac{e^{-h_{ij}\left(s_{ij},\theta_{ij},J\right)}}{z_{ij}\left(\theta_{ij},J\right)}.$$

The complete partition function and multiplex probability can therefore be obtained as products of pair-specific quantities, where each multilink can be regarded as a configuration of a Curie–Weiss system. To obtain an explicit expression for $z_{ij}\left(\theta_{ij},J\right)$, we use a Hubbard–Stratonovich transformation and the Laplace theorem [49] in the limit M→∞. The details are provided in Appendix A and are a generalization of the approach used in [50]. The final result is

(52) $$z_{ij}\left(\theta_{ij},J\right)=2^{M}e^{-\frac{M}{2}\theta_{ij}-2JMu_{ij}\left(u_{ij}-1\right)}\cosh^{M}\left(2Ju_{ij}-\frac{\theta_{ij}}{2}\right),$$

where $u_{ij}$ is the solution to the equation

(53) $$u_{ij}=\frac{1}{2}+\frac{1}{2}\tanh\left(2Ju_{ij}-\frac{\theta_{ij}}{2}\right).$$

The solutions to the above equation will be discussed in the next section.

Now, given a particular real multiplex network $\vec{G}*$, the log-likelihood, as defined, in general, in Equation (21), is

(54) $$L\left(\vec{\theta },J\right)=\ln P(\vec{G}*,\vec{\theta },J)=\sum_{i<j}\left[-h_{ij}\left(s_{ij}^{*},\theta_{ij},J\right)-\ln z_{ij}\left(\theta_{ij},J\right)\right].$$

At a stationary point of L, the derivatives of L with respect to every Lagrange multiplier must equal zero. As we show in Appendix B, this leads to the maximum likelihood equations

(55) $$\sum_{j\neq i}^{N}\sum_{\alpha =1}^{M}{g_{ij}^{*}}^{\alpha }=M\sum_{j\neq i}^{N}u_{ij}^{*}\;\forall i$$

(56) $$\frac{4}{M}\sum_{i<j}\sum_{\alpha <\beta }{g_{ij}^{*}}^{\alpha }{g_{ij}^{*}}^{\beta }=2M\sum_{i<j}{\left(u_{ij}^{*}\right)}^{2}$$

where $u_{ij}^{*}$, being the solution to Equation (53) with $\left(\vec{\theta },J\right)$ replaced by $\left(\vec{\theta }*,J^{*}\right)$, is implicitly related to the maximum likelihood parameters $\left(\vec{\theta }*,J^{*}\right)$. Note that the quantities on the LHS of Equations (55) and (56) are precisely the quantities that we constrained from the start, namely, $M{\bar{k}}_{i}^{*}$ and $4O^{*}/M$, respectively. According to the maximum likelihood principle, these empirical quantities must equal their respective ensemble averages, $M{\langle {\bar{k}}_{i}\rangle }_{\theta^{*},J^{*}}$ and $4{\langle O\rangle }_{\theta^{*},J^{*}}/M$, which appear on the RHS. The quantity $u_{ij}^{*}$ can therefore be considered as an *average* probability of a link occurring between the nodes *i* and *j*, which is equal throughout the *M* layers and is, therefore, a measure of the density of links in the multilink $m_{ij}$. This is similar to how we identified $p_{ij}$ to be the connection probability in the ACM, which was based solely on the constraints ${\bar{k}}_{i}$. In support of this idea, we see that, in the case $J^{*}=0$, the Lagrange multipliers $\vec{\theta }*$ reduce it to the value ${\vec{\theta }}^{0}\equiv \vec{\theta }*{|}_{J^{*}=0}$, such that

(57) $${\left.u_{ij}^{*}\right|}_{J^{*}=0}=\frac{1}{2}\left[1+\tanh\left(-\frac{\theta_{i}^{0}+\theta_{j}^{0}}{2}\right)\right]=\frac{e^{-(\theta_{i}^{0}+\theta_{j}^{0})}}{1+e^{-(\theta_{i}^{0}+\theta_{j}^{0})}}=p_{ij}\left({\vec{\theta }}^{0}\right)$$

which is identical to the expression in Equation (29), providing the link probability $p_{ij}$ obtained in Section 2.5 in the absence of the constraint for the overlap. The quantity $u_{ij}^{*}$ can therefore possibly be interpreted as a *mean-field* quantity that *globally* incorporates the layer interdependence that was introduced through the overlap $O^{*}$, but *locally* treats the layers as if they were independent. A characteristic of mean-field theories is that the effects of all elements of a system on a given element are approximated by a single, average effect.

Formally, we can calculate the entropy of the data, given the model, as the maximized likelihood using Equations (25) and (54):

(58) $$\begin{array}{ccc} S(\vec{\theta }*,J^{*}) & = & -L(\vec{\theta }*,J^{*})\\ & = & -\ln P(\vec{G}*,\vec{\theta }*,J^{*})\\ & = & H\left(\vec{G}*,\vec{\theta }*,J^{*}\right)+\sum_{i<j}\ln z_{ij}\left(\theta_{ij}^{*},J^{*}\right)\\ & = & M\sum_{i=1}^{N}\theta_{i}^{*}{\bar{k}}_{i}^{*}-\frac{4J^{*}}{M}O^{*}+\sum_{i<j}\ln z_{ij}\left(\theta_{ij}^{*},J^{*}\right), \end{array}$$

which requires the knowledge of the parameters $\vec{\theta }*$ and $J^{*}$ (which are, however, defined only implicitly through $u_{ij}^{*}$). Comparing the above expression with Equation (37), we see that $S\left({\vec{\theta }}^{0},0\right)=S_{0}\left({\vec{\theta }}^{0}\right)$, as expected, i.e., the model with $J^{*}=0$ has the same entropy as the equivalent ACM with no overlap, for the same value of ${\vec{\theta }}^{0}$. Similarly, $L\left({\vec{\theta }}^{0},0\right)=L_{0}\left({\vec{\theta }}^{0}\right)$ for the maximized likelihood in the two models. In order to understand the relationship between the entropies of the two models when $J^{*}\neq 0$, let us first note that a positive (resp. negative) coupling strength $J^{*}$ means that the empirical overlap $O^{*}$ is larger (resp. smaller) than the expected overlap under the null model with $J^{*}=0$, i.e.,

(59) $$O^{*}≶{\langle O\rangle }_{{\vec{\theta }}^{0}}\;\Leftrightarrow \;J^{*}≶0$$

where we have used the notation in Equation (36). However, one should not naively conclude from the combination of Equations (58) and (59) that the entropy of the model with $J^{*}<0$ is larger than the entropy of the model with $J^{*}=0$, because the two partition functions are different, and also because the two entropies are calculated for different Lagrange multipliers, i.e., ${\vec{\theta }}^{*}\neq {\vec{\theta }}^{0}$ when $J^{*}\neq 0$. In fact, we can actually show that the entropy of the model with $J^{*}\neq 0$ is always smaller than the one for the model with $J^{*}=0$. To see this, we introduce the relative entropy (or Kullback–Leibler divergence) between the two models, as follows:

(60) $$R\left({\vec{\theta }}^{0},\vec{\theta }*,J^{*}\right)\equiv \sum_{\vec{G}\in G_{N}^{M}}P(\vec{G},\vec{\theta }*,J^{*})\ln\frac{P(\vec{G},\vec{\theta }*,J^{*})}{P_{0}\left(\vec{G},{\vec{\theta }}^{0}\right)}\geq 0,$$

where the last inequality is a well-known property of the relative entropy, and the equality is realized if, and only if, $P_{0}\left(\vec{G},{\vec{\theta }}^{0}\right)$ and $P(\vec{G},\vec{\theta }*,J^{*})$ are identical, which, in turn, requires $J^{*}=0$, yielding ${\vec{\theta }}^{0}=\vec{\theta }*$ and $R({\vec{\theta }}^{0},{\vec{\theta }}^{0},0)=0$. For $J^{*}\neq 0$, we can write

(61) $$\begin{array}{ccc} R({\vec{\theta }}^{0},\vec{\theta }*,J^{*}) & = & \sum_{\vec{G}\in G_{N}^{M}}P(\vec{G},{\vec{\theta }}^{*},J^{*})\ln P(\vec{G},\vec{\theta }*,J^{*})-\sum_{\vec{G}\in G_{N}^{M}}P(\vec{G},\vec{\theta }*,J^{*})\ln P_{0}\left(\vec{G},{\vec{\theta }}^{0}\right)\\ & = & -S\left(\vec{\theta }*,J^{*}\right)+\sum_{\vec{G}\in G_{N}^{M}}P(\vec{G},\vec{\theta }*,J^{*})\left[H_{0}\left(\vec{G},{\vec{\theta }}^{0}\right)+\ln Z_{0}\left({\vec{\theta }}^{0}\right)\right]\\ & = & -S\left(\vec{\theta }*,J^{*}\right)+\sum_{\vec{G}\in G_{N}^{M}}P_{0}\left(\vec{G},{\vec{\theta }}^{0}\right)\left[H_{0}\left(\vec{G},{\vec{\theta }}^{0}\right)+\ln Z_{0}\left({\vec{\theta }}^{0}\right)\right]\\ & = & -S\left(\vec{\theta }*,J^{*}\right)+\sum_{\vec{G}\in G_{N}^{M}}P_{0}\left(\vec{G},{\vec{\theta }}^{0}\right)\ln P_{0}\left(\vec{G},{\vec{\theta }}^{0}\right)\\ & = & -S\left(\vec{\theta }*,J^{*}\right)+S_{0}\left({\vec{\theta }}^{0}\right), \end{array}$$

where we have used the fact that $H_{0}\left(\vec{G},{\vec{\theta }}^{0}\right)=M\sum_{i=1}^{N}\theta_{i}^{0}\;{\bar{k}}_{i}\left(\vec{G}\right)$ has the same expectation value, equal to $M\sum_{i=1}^{N}\theta_{i}^{0}\;{\bar{k}}_{i}\left(\vec{G}*\right)$, under both $P(\vec{G},\vec{\theta }*,J^{*})$ and $P_{0}\left(\vec{G},{\vec{\theta }}^{0}\right)$:

(62) $$\begin{array}{ccc} \sum_{\vec{G}\in G_{N}^{M}}P(\vec{G},\vec{\theta }*,J^{*})H_{0}\left(\vec{G},{\vec{\theta }}^{0}\right) & = & M\sum_{i=1}^{N}\theta_{i}^{0}\left[\sum_{\vec{G}\in G_{N}^{M}}P(\vec{G},\vec{\theta }*,J^{*}){\bar{k}}_{i}\left(\vec{G}\right)\right]\\ & = & M\sum_{i=1}^{N}\theta_{i}^{0}\;{\bar{k}}_{i}\left(\vec{G}*\right)\\ & = & M\sum_{i=1}^{N}\theta_{i}^{0}\left[\sum_{\vec{G}\in G_{N}^{M}}P_{0}\left(\vec{G},{\vec{\theta }}^{0}\right){\bar{k}}_{i}\left(\vec{G}\right)\right]\\ & = & \sum_{\vec{G}\in G_{N}^{M}}P_{0}\left(\vec{G},{\vec{\theta }}^{0}\right)H_{0}\left(\vec{G},{\vec{\theta }}^{0}\right). \end{array}$$

Now, applying the inequality $R({\vec{\theta }}^{0},\vec{\theta }*,J^{*})\geq 0$ in Equation (60) to Equation (62), we get

(63) $$0\leq S\left(\vec{\theta }*,J^{*}\right)\leq S_{0}\left({\vec{\theta }}^{0}\right),$$

confirming that the entropy of the model with $J^{*}\neq 0$ is always smaller than the one for the model with $J^{*}=0$, consistent with the fact that the former is more constrained than the latter.

## 4. Local Phase Transitions in the Model

**Figure 1:**
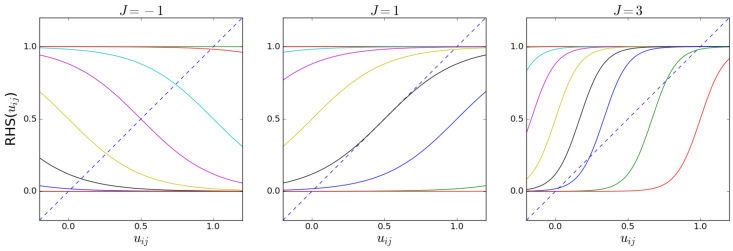
A graphical illustration of the solution(s) of Equation (53). The solid lines show the RHS of Equation (53) as a function of $u_{ij}$ for the different parameters $\theta_{ij}\in \left\{-12,-8,-4,-2,0,2,4,8,12\right\}$, while the dashed line shows the LHS, which equals $u_{ij}$ itself. For a given parameter value, the solutions of Equation (53) are the intersection between the dashed and the corresponding solid line. Each panel corresponds to a different value of *J* (in the rest of the paper, we will consider only J≥0).

The number of solutions of Equation (53) depends on the values of the parameters $\theta_{ij}=\theta_{i}+\theta_{j}$ and *J*. We illustrate this fact in Figure 1, where both the LHS and the RHS of Equation (53) are plotted as a function of $u_{ij}$ for various values of $\theta_{ij}$ and *J*. The appearance of multiple solutions signals the existence of *phase transitions* in the limit when the number *M* of layers diverges, which determine abrupt changes in the value of $u_{ij}$ and, therefore, also in the properties of the multilink $m_{ij}$ and the structure of the multiplex as a whole. The configurations for $m_{ij}$ that are separated by a phase transition are the *phases* of the multilink. The point where multiple solutions appear or vanish is the *bifurcation point*.

Figure 1 shows that, at the interval $0\leq u_{ij}\leq 1$, there can be either one, two, or three solutions, and that for $\theta_{ij}\to +\infty$ or $\theta_{ij}\to -\infty$ there is always one solution, namely, $u_{ij}=0$ or $u_{ij}=1$, respectively. The number of solutions depends on whether the slope (derivative) of the RHS (which depends on the parameters) exceeds the slope of the LHS (which is always equal to 1) of Equation (53) at their intersection. From now on, we will consider only the case J≥0, which corresponds to a tendency to create an increased inter-layer overlap compared with the model with J=0. The case J<0 corresponds to the opposite case where the overlap is suppressed, which we do not discuss here. New solutions appear or vanish at the point where Equation (53) is satisfied *and* the derivatives of the LHS and RHS of Equation (53) are equal:

(64) $$1=J\left[1-\tanh^{2}\left(2Ju_{ij}-\frac{\theta_{ij}}{2}\right)\right].$$

**Figure 2:**
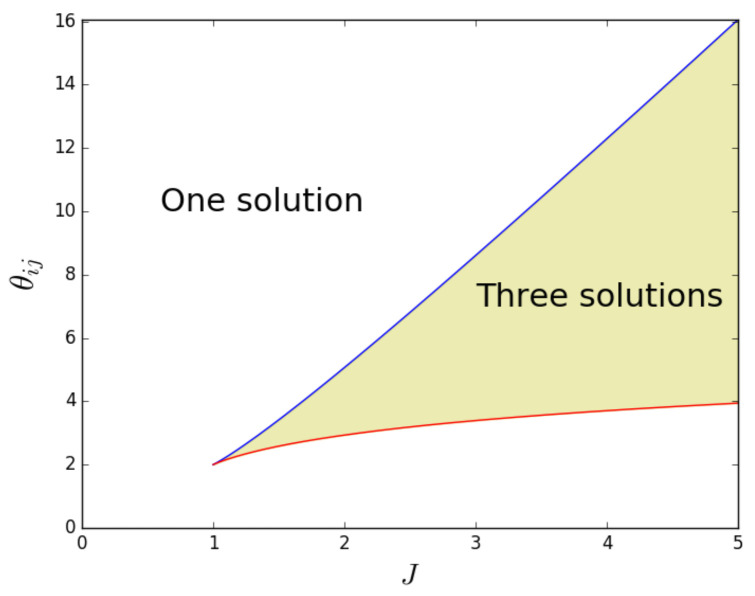
The upper (blue) and lower (red) curves correspond to Equations (65) and (66), respectively, which delimit the region of phase space (yellow area), for which Equation (53) has three solutions. Note that the ‘zero-field’ condition $\theta_{ij}=2J$ is always in the yellow area when J>1, so the condition J>1 is sufficient to ensure that the system in zero field is in the magnetized (symmetry-broken) phase.

Equation (64) cannot be satisfied if 0≤J≤1, since $0\leq \tanh^{2}\left(x\right)<1$ for x∈R, and, therefore, if J≤1, a phase transition is impossible, and there is a unique solution for $u_{ij}$. When J>1, Equation (64) gives us two potential solution branches, $u_{ij}^{\pm }=\frac{1}{2}\pm \frac{1}{2}\sqrt{1-1/J}$, where we have used $2u_{ij}-1=\tanh\left(2Ju_{ij}-\theta_{ij}/2\right)$. Equation (53) can be written as $\theta_{ij}=4Ju_{ij}-\ln\left[u_{ij}/\left(1-u_{ij}\right)\right]$ using the identity $\tanh^{-1}x=\frac{1}{2}\ln\left[\left(1+x\right)/\left(1-x\right)\right]$. By then substituting $u_{ij}^{\pm }$ into this expression for $\theta_{ij}$, we obtain the equations for the two curves in the $\left(J,\theta_{ij}\right)$ plane that mark the points where additional solutions appear or vanish:

(65) $$\theta_{ij}^{+}\left(J\right)=\frac{2\sqrt{J}}{\sqrt{J}-\sqrt{J-1}}-\ln\left(\frac{\sqrt{J}+\sqrt{J-1}}{\sqrt{J}-\sqrt{J-1}}\right),$$

(66) $$\theta_{ij}^{-}\left(J\right)=\frac{2\sqrt{J}}{\sqrt{J}+\sqrt{J-1}}-\ln\left(\frac{\sqrt{J}-\sqrt{J-1}}{\sqrt{J}+\sqrt{J-1}}\right),$$

as shown in Figure 2. In the region between the two curves, there are three solutions to Equation (53). Note that the ‘zero-field’ condition $\theta_{ij}=2J$ is always in that region when J>1. This means that the condition J>1 is sufficient to ensure that the system is in the magnetized (symmetry-broken) phase when in the absence of the external field. However, when $\theta_{ij}\neq 2J$, the condition J>1 is necessary but not sufficient. In particular, generally, it may happen that, for a given value of J>1, different pairs of nodes will be in different (magnetized or non-magnetized) phases depending on the value of $\theta_{ij}$. This shows that the system can undergo a multitude of separate phase transitions if the parameters $\left\{\theta_{ij}\right\}$ remain fixed and *J* is varied.

**Figure 3:**
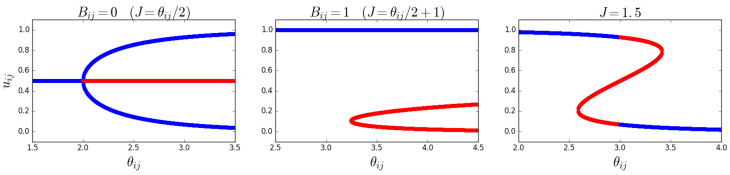
Solutions for $u_{ij}$ as a function of $\theta_{ij}$ for different parameter values. The blue and red segments of the curve(s) correspond to the stable and unstable solutions of Equation (53), respectively. **Left** panel: $B_{ij}=0$ (with *J* varying accordingly). **Middle** panel: $B_{ij}=1$ (with *J* varying accordingly). **Right** panel: constant value of J=1.5, which translates to a non-constant $B_{ij}$.

In the magnetized phase, the phenomenon of symmetry breaking will occur: the typical realized values of the ‘magnetization’ will not coincide with the corresponding ensemble average. In the zero-field case ($\theta_{ij}=2J$), the symmetry breaking is ‘spontaneous’, i.e., not induced by any field pointing in a preferred direction, while in the nonzero-field case, the symmetry is broken by the field itself. This well-known property of the Ising model has specific implications for our problem here. Indeed, while certain values of $\theta_{ij},J$ may solve the maximum likelihood Equations (55) and (56), the corresponding solutions to Equation (53) may not necessarily maximize the likelihood, and are therefore not ‘valid’ (or *stable*). Once the values $\theta_{ij}^{*}$ and $J^{*}$ that solve the maximum likelihood equations are found, the graph probability corresponding to this set of values can be written as a function of the configuration of the graph (or the collection of configurations of the multilinks $m_{ij}$), and one can check which typical configurations (those minimizing the Hamiltonian) arise. As Figure 1 suggests, in the regime where there are three solutions, $u_{ij}$, one value will be relatively high (which corresponds to a relatively high density of links in $m_{ij}$), another value will be relatively low (which corresponds to a relatively low density of links in $m_{ij}$), and the third value will be between the other two, corresponding to an intermediate density of links in $m_{ij}$. By inspecting the (pair) Hamiltonian in Equation (47) in terms of the $\sigma_{ij}^{\alpha }=2g_{ij}^{\alpha }-1$ variable, it becomes clear which of the three solutions $u_{ij}^{*}$ are viable (stable). In the case where $B_{ij}=0$, or equivalently, when $\theta_{ij}=2J$, the (pair) Hamiltonian is symmetric with respect to a change in sign, $\sigma_{ij}^{\alpha }\to -\sigma_{ij}^{\alpha }$, which means that the high- and low-density solutions are equal. This is the symmetry-broken situation we have discussed in Section 3.1. In this case, the intermediate-density solution will result in a lower value for the Hamiltonian than the high- and low-density solutions. The viable (stable) solutions are therefore the high- and low-density ones. In the case where $B_{ij}\neq 0$, it is clear that the high-density solution minimizes the Hamiltonian when $B_{ij}>0$ and maximizes it when $B_{ij}<0$. The low-density solution minimizes the Hamiltonian when $B_{ij}<0$ and maximizes it when $B_{ij}>0$. The intermediate solution will, however, never minimize the Hamiltonian when B≠0, and is therefore never viable (stable). From these considerations, it becomes clear that a phase transition, corresponding to a sudden change in $u_{ij}$, may only happen when we cross from a negative (positive) $B_{ij}$ to a positive (negative) $B_{ij}$ (when J>1). Figure 3 shows the symmetric stable solutions $u_{ij}$ in the case where $B_{ij}=0$, with the bifurcation occurring at J=1. In case of the positive field $B_{ij}=+1$, it shows a single stable solution curve, which is the high-density solution (in the case where $B_{ij}=-1$, this image would be flipped with respect to the $u_{ij}^{*}=1/2$ axis). The right panel in Figure 3 shows that the value of the stable solution $u_{ij}$ jumps when $B_{ij}$ crosses from positive to negative, as expected.

Combining the above considerations for all multilinks simultaneously, and adding the other constraint on the layer-averaged degrees, the multiplex will undergo a sequence of phase transitions, determining a hierarchy of increasingly ordered (magnetized, or rather ‘multiplexed’ in this case) phases where, for an increasing number of pairs of nodes, the links in different layers will tend to ‘align’ to each other (for J>1). The separations between these phase transitions will depend on the values of the enforced layer-averaged degrees, which determine $\vec{\theta }*$. The fully ordered phase, where all pairs of nodes are multiplexed, is the one where all the *M* layers of the multiplex are perfectly aligned, and are, therefore, basically an identical copy of each other. We might say that, in this case, the effective number of independent layers is $M_{\mathrm{eff}}\approx 1$, and the expected overlap is maximal and proportional to the expected number ${\langle L\rangle }_{\vec{\theta }*,J^{*}}=\sum_{\alpha =1}^{M}\sum_{i<j}u_{ij}^{*}$ of links in the entire multiplex:

(67) $${\langle O\rangle }_{\vec{\theta }*,J^{*}}\approx \sum_{\alpha <\beta }\sum_{i<j}u_{ij}^{*}=\left(M/2\right){\langle L\rangle }_{\vec{\theta }*,J^{*}},$$

since ${\langle g_{ij}^{\alpha }g_{ij}^{\beta }\rangle }_{\vec{\theta }*,J^{*}}\approx {\langle g_{ij}^{\alpha }\rangle }_{\vec{\theta }*,J^{*}}=u_{ij}^{*}$ for most pairs, i.e., α,β, of layers. In the opposite extreme, we have a fully disordered phase where no pair of nodes is multiplexed (for instance, if J<1), so the effective number of independent layers is maximal ($M_{\mathrm{eff}}\approx M$), and the expected overlap is basically of the order of that given by Equation (36) for the model with $J^{*}=0$, i.e.,

(68) $${\langle O\rangle }_{\vec{\theta }*,J^{*}}\approx \sum_{\alpha <\beta }\sum_{i<j}{\left(u_{ij}^{*}\right)}^{2},$$

since ${\langle g_{ij}^{\alpha }g_{ij}^{\beta }\rangle }_{\vec{\theta }*,J^{*}}\approx {\langle g_{ij}^{\alpha }\rangle }_{\vec{\theta }*,J^{*}}{\langle g_{ij}^{\beta }\rangle }_{\vec{\theta }*,J^{*}}={\left(u_{ij}^{*}\right)}^{2}$ for most pairs of layers. The relationship between ${\langle O\rangle }_{\vec{\theta }*,J^{*}}$ and ${\langle L\rangle }_{\vec{\theta }*,J^{*}}$ will depend on the specific values of ${\left\{u_{ij}^{*}\right\}}_{i<j}$, so ultimately, on the enforced degree sequence. Between these two extremes, if the phases are well separated (which here means that the enforced degrees of different nodes have very different values), there will be intermediate regimes where ${\langle O\rangle }_{\vec{\theta }*,J^{*}}$ and ${\langle L\rangle }_{\vec{\theta }*,J^{*}}$ scale in a way that is between the two limiting scalings. All these general considerations will be confirmed in the next sections with numerical, analytical, and empirical analyses.

## 5. Numerical Analysis

Equations (53), (55), and (56) are the key equations of our OACM model. These equations are generally, however, very difficult to solve. Therefore, before creating a null model for a real-world network by solving the maximum likelihood equations to find the Lagrange multipliers, we shall first treat the Lagrange multipliers as free parameters in order to explore and analyze the properties of the model as a function of these parameters. This analysis shall be performed by utilizing the Metropolis–Hastings algorithm [51]. This algorithm can be used to sample the exponential probability distribution defined by the Hamiltonian of the model. By sampling the distribution, we numerically obtain various properties of the graph ensemble, which may then be compared to our analytical results in order to test the validity of the latter. Note that the sampling of the exponential distribution defined by a specific Hamiltonian may also be regarded as the simulation of a multiplex that corresponds to that Hamiltonian.

### 5.1. Exploring the Parameter Space

In order to explore the space of parameters, we are primarily interested in the difference between statistically *homogeneous* networks and statistically *heterogeneous* ones. To this end, we will explore the parameter space $\left(\theta_{1},\ldots ,\theta_{N},J\right)$ of the model by specifying a value for *J* and sampling certain transformed parameters $x_{1},\ldots ,x_{N}$ from a distribution for each class, where $x_{i}\equiv e^{-\theta_{i}}$. The quantity $x_{i}$ will be referred to as the ‘fitness’, or ‘hidden variable’, of node *i*. The broader the distribution of the fitness, the more heterogeneous the resulting network structure.

#### 5.1.1. Homogeneous Fitness: Erdős–Rényi Graphs with Overlap

The simplest distribution from which we can sample $x_{1},\ldots ,x_{N}$ is the delta distribution centered at *x*, such that $x_{1}=x_{2}=\ldots =x_{N}\equiv x$ and, therefore, $\theta_{1}=\theta_{2}=\ldots =\theta_{N}\equiv \theta =-\ln x$, resulting in statistically homogeneous networks. With this choice of parameters, our model is an extension of the Erdős–Rényi model, which is a random graph model that can be derived within the ERGM by solely constraining the total number of links in the network, and where all links occur with the same probability. As we shall see, the extension derives from the fact that the extra constraint on the overlap can lead to a symmetry-breaking phase transition, although the broken symmetry might not manifest at first sight. Indeed, since the parameters are the same for all pairs of nodes, the condition for the existence of multiple solutions is also the same, and, therefore, there is a unique phase transition where, depending on the values of θ and *J*, pairs of nodes are either all ‘magnetized’ or all ‘non-magnetized’. Similarly, since here $\theta_{ij}=\theta_{i}+\theta_{j}=2\theta$ ∀i,j, the spontaneous symmetry-breaking condition discussed in Section 3.1 for the vanishing of the external field is the same for all pairs of nodes, and given by J=θ. In the symmetry-broken (magnetized) phase, for all pairs of nodes, the expected value of $\sum_{\alpha =1}^{M}g_{ij}^{\alpha }$ (or equivalently, of the ‘magnetization’ $\sum_{\alpha =1}^{M}\sigma_{ij}^{\alpha }$) is the same, and is always between the two typical (high-density and low-density) realized values. However, since all pairs are independent, the actual realized values of $\sum_{\alpha =1}^{M}g_{ij}^{\alpha }$ are also independent across pairs, so on average, over the entire network, the magnetization will realize both the low-density and high-density values, with equal probability. In other words, different pairs of nodes are i.i.d. realizations of the same system. This is a peculiar situation where the realized values of *L* and *O* (which represent sums of all pairs of nodes) will still coincide with their expected values as if no symmetry breaking was present, even if different pairs of nodes actually realize different symmetry-broken values that are individually different from the expected value. The net result is an expected number of links 〈L〉=MN(N−1)/4) equal to half the maximum one, or equivalently, an average zero magnetization in the associated spin system. Similar considerations apply to the case J≠θ, with the difference that, in that case, the symmetry is not broken spontaneously, but by the direction of the external field (value of θ), which implies that the two typical realized values of the magnetization for a given pair of nodes are no longer symmetric around the expected value. Still, both typical values will be realized, independently and with their probabilities, across the entire network, because different pairs of nodes are still independent. So, irrespective of the value of *J* and θ, we expect to observe realized values of *L* and *O* that correspond again to what one would observe without symmetry breaking, using the ensemble averages for each pair, irrespective of the phase of the system. All these considerations are confirmed below.

By looking at Equation (38), we can see that a uniform θ essentially means that instead of constraining the average layer degrees ${\bar{k}}_{i}$, we constrain the total number of links *L* in the multiplex network. In this case, the combined maximum entropy and maximum likelihood equations become

(69) $$u=\frac{1}{2}+\frac{1}{2}\tanh\left(2J^{*}u-\theta^{*}\right)$$

(70) $$\sum_{i<j}^{N}\sum_{\alpha =1}^{M}{g_{ij}^{*}}^{\alpha }=\frac{MN(N-1)}{2}u^{*}={\langle L\rangle }_{\theta^{*},J^{*}}$$

(71) $$\frac{4}{M}\sum_{i<j}\sum_{\alpha <\beta }{g_{ij}^{*}}^{\alpha }{g_{ij}^{*}}^{\beta }=MN\left(N-1\right){\left(u^{*}\right)}^{2}=\frac{4}{M}{\langle O\rangle }_{\theta^{*},J^{*}}$$

where $u^{*}=u\left(\theta^{*},J^{*}\right)$ is the solution to Equation (69). Note that we now have a single equation for *u*, confirming the existence of a single *global* phase transition across the multiplex network, rather than separate local phase transitions for every multilink $m_{ij}$. Additionally, we note that if $u^{*}$ can be considered as the density (and the link probability) of the network, then the value of $u^{*}$ is exactly the same as the value of the density *p* in the Erdős–Rényi model [14,27], which solely constrains the number of links in the network. The difference between our model and the Erdős–Rényi model is that our model contains the possibility of a phase transition. However, since the number of links 〈L〉 also determines the overlap 〈O〉, the two quantities cannot be tuned independently of each other.

**Figure 4:**
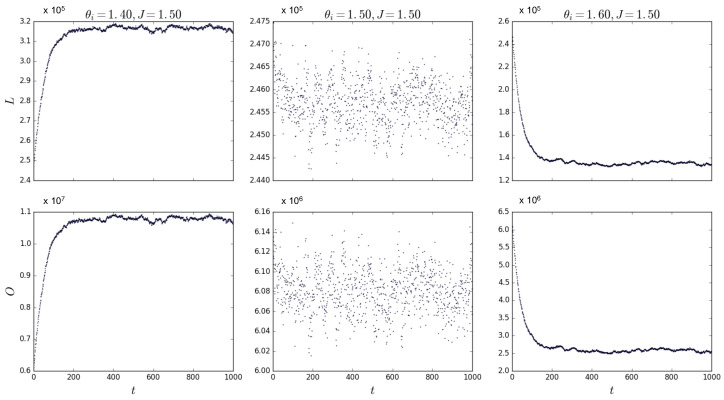
Total number of links *L* (top panels) and inter-layer overlap *O* (bottom panels) as a function of simulation time using the Metropolis–Hastings algorithm for J=1.5, N=100, M=100. **Left** panels: θ=1.4. **Middle** panels: θ=1.5=J (symmetry-broken case). **Right** panels: θ=1.6. For fixed *J*, varying θ determines a phase transition from a high-density phase to a low-density phase.

By using the Metropolis–Hastings algorithm, we have sampled our ERGM for multiplexes with M=100 layers and N=100 nodes for various values of θ and/or *J*. If we repeat the simulations for J=1.5 and θ=1.4, θ=1.5, and θ=1.6, the system must undergo a phase transition as per Figure 3. We expect an abrupt change in the value of $u^{*}$, and according to Equations (70) and (71), we therefore expect an abrupt change in the equilibrium value of both *L* and *O*. Figure 4 shows simulations for θ∈{1.4,1.5,1.6} confirming the transition from a relatively high to a low density as the value of the field B=J−θ changes sign. These simulations have been repeated for different combinations of values for *J* and θ around the point where *B* changes sign, confirming the results shown here. Note that the middle plot in Figure 4 shows that the algorithm converges to multiplexes with a density of 1/2, confirming that, when B=0, *L* is approximately half of the total amount of possible links in the multiplex, as we expected above.

**Figure 5:**
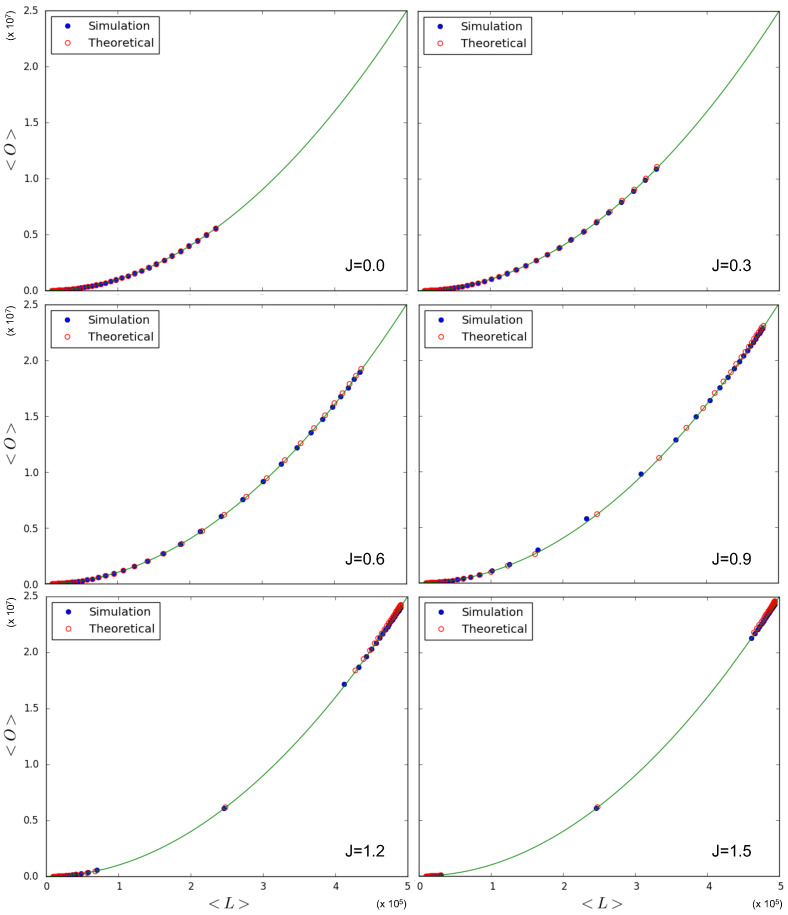
Relationship between the expected inter-layer overlap 〈O〉 and the total number of links 〈L〉 in homogeneous multiplexes with N=100, M=100, and $\theta_{i}=\theta$ for all i=1,N. The blue points correspond to simulations obtained via the Metropolis–Hastings algorithm for J∈{0.0,0.3,0.6,0.9,1.2,1.5} and θ∈[0.05,2.00] in steps of Δθ=0.05. The open red circles are the corresponding theoretically predicted points. The solid curve corresponds to the quadratic trend $\langle O\rangle ={\langle L\rangle }^{2}/N^{2}$ predicted for all J≥0. Multiple solutions for $u_{ij}^{*}$ first appear when J>1, but the system keeps following the quadratic trend, albeit drifting away from the central point obtained for the zero-field case θ=J (corresponding to a spontaneously broken symmetry).

In Figure 5 we test the prediction, given by Equations (70) and (71), of the quadratic relationship $\langle O\rangle ={\langle L\rangle }^{2}/N^{2}$. Note that this quadratic trend is predicted irrespective of the value of J>0, and even coincides with what Equation (68) predicts in the case J=0 for a homogeneous multiplex with constant θ, as considered here. So, in this case, the expected relationship between 〈O〉 and 〈L〉 is not informative regarding the phase transition, although the specific values picked up by the system along the curve are. Indeed, we again simulate multiplexes with M=100 layers, N=100 nodes, and a variety of values for θ and *J*. Each simulation results in a value for 〈L〉 and a value for 〈O〉, which we plot against each other. These points are then compared to the theoretical points predicted by Equations (69)–(71) for the chosen parameter values, and added to Figure 5. We see that the relationship between simulated quantities is in agreement with the one predicted by the model. As we had anticipated, this is the result of the fact that different pairs of nodes are i.i.d. realizations of the same system, so that the ensemble average is realized as a sample average of the pairs of nodes across the network, even if in the symmetry-broken phase, the ensemble average of $\sum_{\alpha =1}^{M}g_{ij}^{\alpha }$ is not representative of any of the values realized locally for individual pairs of nodes. Therefore, the only scaling we observe coincides with the one given in Equation (68) for the ‘non-magnetized’ regime in the case where θ is the same for all nodes. The only, although very important, signature of the phase transition we see in Figure 5 is the fact that, for J>1 and θ≠J, both the simulated data and the corresponding theoretical predictions ‘drift away’ from the intermediate values of 〈L〉 (which are still obtained for θ=J) towards either low (θ>J) or high (θ<J) values of 〈L〉. This is because the realized multiplex networks are either low-density or high-density, which is an indication of a phase transition occurring when increasing the value of *J*, exactly as predicted by Figure 3.

We conclude our discussion of the homogeneous case by noting that, given an empirical multiplex $\vec{G}*$ of interest, the entropy of the data given, in general, by Equation (58) reduces, in this case, to

(72) $$\begin{array}{ccc} S(\theta^{*},J^{*}) & = & M\theta^{*}\sum_{i=1}^{N}{\bar{k}}_{i}^{*}-\frac{4J^{*}}{M}O^{*}+\sum_{i<j}\ln z_{ij}\left(2\theta^{*},J^{*}\right)\\ & = & 2\theta^{*}L^{*}-\frac{4J^{*}}{M}O^{*}+\frac{N(N-1)}{2}\ln z(2\theta^{*},J^{*}), \end{array}$$

where we have used Equation (9) (denoting, via $L^{*}=L\left(\vec{G}*\right)$, the total number of links in the multiplex, which also equals the expected value ${\langle L\rangle }_{\theta^{*},J^{*}}$) and the fact that the pair partition function $z_{ij}$, given by Equation (52), has the same value $z\left(2\theta^{*},J^{*}\right)\equiv z_{ij}\left(2\theta^{*},J^{*}\right)$ for all the N(N−1)/2 pairs of nodes. From Equation (72), we see that the entropy is determined, as expected, by both $L^{*}$ and $O^{*}$. At the same time, we know that $O^{*}$ depends uniquely and quadratically on $L^{*}$ in this homogeneous model. The values achieved by the entropy are, therefore, bound by the relationship between $L^{*}$ and $O^{*}$, which here is the same irrespective of the value of $J^{*}$, including when $J^{*}=0$. In any case, the entropy also depends on the specific values of $\left(\theta^{*},J^{*}\right)$, and Equation (63) guarantees that an upper bound for $S(\theta^{*},J^{*})$ is given by the entropy $S_{0}\left(\theta^{0}\right)$ of the ACM model with $J^{*}=0$ and $\theta^{*}=\theta^{0}$ (clearly, the homogeneity implies that $\theta_{i}^{0}=\theta^{0}$ for all i=1,N in the ACM model as well).

#### 5.1.2. Power-Law-Distributed Fitness: Scale-Free Networks with Overlap

We now move away from the homogeneous case and consider a situation where the fitness values ${\left\{x_{i}\right\}}_{i=1}^{N}$ are drawn from a heavy-tailed distribution, in particular, a power law. This choice will produce a high degree of heterogeneity. In the ACM (see Section 2.5), the expected degree distribution is determined by the Lagrange multipliers $\theta_{i}$, or equivalently, the transformed hidden variables $x_{i}=e^{-\theta_{i}}$. If *x* is distributed according to a power law, the expected degree distribution shall be distributed according to a power law as well, with the modulo as an upper cut-off. Since our OACM is an extension of the ACM, we will still sample $x_{i}$ from a power law distribution $P\left(x\right)∼x^{-\gamma }$ for various values of γ, even though the expected degree distribution is not solely determined by the hidden variables $\left\{x_{i}\right\}$, but depends on *J* as well. In any case, a higher level of heterogeneity in the hidden variables $x_{i}$ will lead to a higher level of heterogeneity in the degrees. Since the parameter space is rather large (N+1-dimensional), we define

(73) $$x_{i}=zx_{0,i}\;$$

where *z* is a scaling factor. We sample $x_{0,i}$ only *once* from every chosen distribution. The value of $x_{i}$ is varied by varying the scaling factor *z*. The parameter space to be explored will then be (z,J), which is 2-dimensional. We deduce that

(74) $$\theta_{i}=-\ln\left(zx_{0,i}\right)\;$$

which shows that an increasing *z* leads to a decreasing $\theta_{i}$. In the ACM, we have shown that the link probability is equal to $p_{ij}=x_{i}x_{j}/\left(1+x_{i}x_{j}\right)$, which means that larger values of $x_{i}$ lead to a larger expected degree, so that increasing all the fitness values will increase the density in the network. This qualitative relationship still holds with the addition of the constraint on the expected overlap (for fixed *J*).

**Figure 6:**
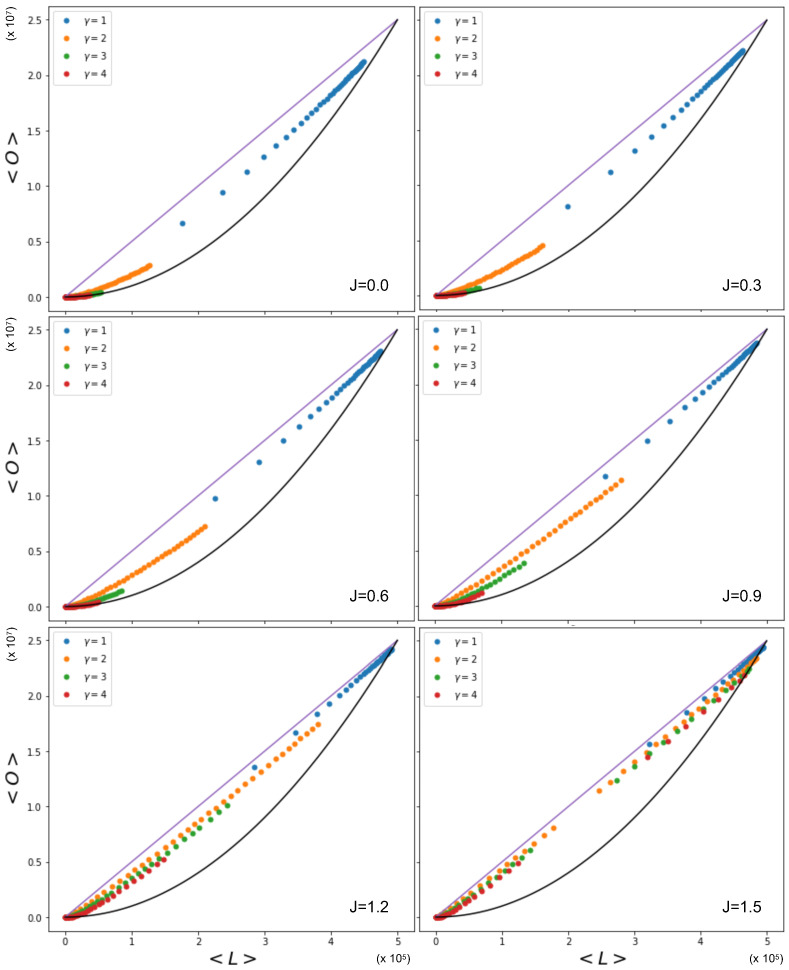
Relationship between the expected inter-layer overlap 〈O〉 and the total number of links 〈L〉 in heterogeneous multiplexes with N=100, M=100, and $x_{0,i}$ sampled from a power law distribution with different values for γ. The colored points correspond to simulations obtained via the Metropolis–Hastings algorithm for J∈{0.0,0.3,0.6,0.9,1.2,1.5} and z∈[0.05,2.00] in steps of Δz=0.05. The straight line corresponds to the upper limit 〈O〉=M〈L〉/2 calculated in Equation (67). The solid curve corresponds to the quadratic trend $\langle O\rangle ={\langle L\rangle }^{2}/N^{2}$ (achieved by homogeneous multiplexes with constant $x_{i}$), which here turns out to mark a lower bound. For increasing values of *J*, and especially as J>1, the system moves closer to the upper bound. For J=1.5, we see that the points are concentrating towards high-density and low-density (symmetry-broken) regimes, drifting away from the intermediate values, like in the homogeneous case. However, this is now the combined result of the behavior of statistically different pairs of nodes, each having a different zero-field condition $\theta_{i}+\theta_{j}=2J$, so the spontaneous symmetry breaking cannot be realized for all node pairs simultaneously.

The complexity of Equations (53), (55), and (56) does not allow us to easily derive the expected relationship between the overlap and the number of links in the network, as was the case when $\theta_{i}$ was constant. It is, however, possible to visualize the relationship between the overlap and the number of links by using the Metropolis–Hastings algorithm. Figure 6 shows this relationship, where $x_{i}$ is sampled from power law distributions with various values of γ, alongside the expected quadratic term previously observed to occur for homogeneous values of the fitness $x_{i}$ (delta distribution). We see that the overlap for a given number of links is higher in the cases where *x* is drawn from a power law distribution than when *x* is drawn from a delta distribution, even though the coupling parameter *J* is kept constant. The cause of this difference lies in the level of heterogeneity of the fitness distribution: unlike the homogeneous case, now different pairs of nodes have very different values of $\theta_{ij}=\theta_{i}+\theta_{j}$, and, therefore, the condition $J=\theta_{ij}/2$ for the vanishing of the ‘external field’ $B_{ij}$ (spontaneous symmetry-breaking condition) cannot be realized simultaneously by all pairs. The figure also shows the effect of different exponents of the power law distributions of the fitness. A smaller value of γ leads to a higher overlap for a given number of links. By increasing the value of γ, the power law distribution becomes more sharply peaked, and will therefore lead to more homogeneous networks. Note, however, that increasing the value of the coupling parameter *J* itself also leads to an increase in the overlap for a given number of links for the same distribution.

Importantly, the phase transition now occurs for different pairs of nodes as *J* is varied. Some pairs of nodes will be in the non-magnetized phase, while others will be in the magnetized phase. The effective number $M_{\mathrm{eff}}$ of independent layers will, in general, depend on the choice of parameters. Among the magnetized pairs, the realized values of the overlap are no longer those corresponding to the ensemble average (as in the homogeneous case), but typically to the symmetry-broken solution with lower energy (hence dictated by the value of $\theta_{ij}$), because no other pair of nodes will, in general, exist with the same parameters and such that the two symmetry-broken values are averaged by the resulting value of the realized overlap. In particular, while for 0<J<1 all node pairs are in the non-magnetized phase, as *J* increases from 1 towards larger values, the pairs of nodes that first undergo the phase transition are the ones with values $\theta_{i}+\theta_{j}$ that fall between the limits set by Equations (65) and (66). As those equations and Figure 2 show, there are more and more combinations $\theta_{i}+\theta_{j}$ entering the magnetized phase as *J* increases. When *J* is sufficiently large, all pairs will be magnetized. Clearly, for any two pairs of nodes, (i,j) and (i,k), that share the same node, *i*, the values of $\theta_{i}+\theta_{j}$ and $\theta_{i}+\theta_{k}$ will be correlated, as they share the same term $\theta_{i}$. This means that the pairs of nodes entering the magnetized phase typically have nodes in common, even if it would be incorrect to say that individual nodes enter the magnetized phase ‘one by one’, while this is certainly correct for individual node pairs, if the sum $\theta_{i}+\theta_{j}$ is different across all of them.

**Figure 7:**
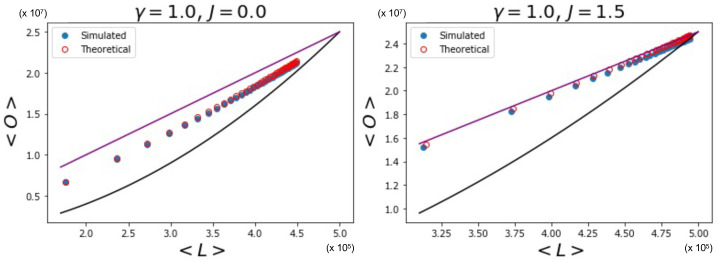
Relationship between the expected inter-layer overlap 〈O〉 and the total number of links 〈L〉 in heterogeneous multiplexes with N=100, M=100, and $x_{0,i}$ sampled from a power law distribution with γ=1. The blue points correspond to simulations obtained via the Metropolis–Hastings algorithm for z∈[0.05,2.00] in steps of Δz=0.05 with J=0 (**left** panel) and J=1.5 (**right** panel). The red open circles are the theoretically predicted values corresponding to the same parameters used in the simulations. The straight line corresponds to the upper limit 〈O〉=M〈L〉/2 calculated in Equation (67). The solid curve corresponds to the quadratic trend $\langle O\rangle ={\langle L\rangle }^{2}/N^{2}$ (achieved by homogeneous multiplexes with constant $x_{i}$), which here turns out to mark a lower bound. We see that, compared with the homogeneous lower bound, the heterogeneity of nodes increases the overlap dramatically, even in the absence of true coupling (J=0). When coupling is present, the overlap is additionally increased and already approaches the upper bound for J=1.5.

Figure 6 indeed shows the effect of the changing number of magnetized node pairs as *J* increases above 1. We note that, for larger and larger *J*, the relationship between 〈O〉 and 〈L〉 tends towards the ‘maximally multiplexed’ linear extreme (shown as a straight line) given in Equation (67). At the same time, we see that the ‘non-multiplexed’ case (J<1) described by Equation (68) now realizes values of the overlap that are very different from the quadratic trend achieved by the homogeneous model (also shown as a solid curve in Figure 6), which now turns out to represent a lower bound. We can ‘zoom in’ to better see this difference by looking at Figure 7, where, by using Equations (53), (55), and (56), we additionally calculate the theoretically predicted values of 〈O〉 and 〈L〉 and compare them to the simulation data, where $x_{0,i}$ is sampled from a power law distribution with γ=1 (the results for γ∈{2,3,4} are qualitatively similar and are therefore not shown here). The figure confirms a strong deviation from the curve for the homogeneous model, even when J=0 (signaling a much higher but spurious overlap, arising only from the rising correlation among node degrees across different layers), and a close agreement with the maximally overlapping value in Equation (67) already for J=1.5 (corresponding to a further increase in overlap, arising from an additional, genuine coupling between layers).

#### 5.1.3. Log-Normally Distributed Fitness

The delta and power law distributions we have considered so far represent examples of completely homogeneous and extremely heterogeneous (especially for γ=1) distributions, respectively. We now consider the log-normal distribution as a third example between these two extremes. This analysis will indeed lead to results that are in some sense intermediate between what we have observed so far, and useful for interpreting the real-world case that we will present later on. A log-normal distribution is the distribution of a random variable whose logarithm is normally distributed (i.e., if the random variable *x* is log-normally distributed, then y=lnx follows a normal distribution). The probability density for a log-normal distribution is

(75) $$P\left(x\right)=\frac{1}{x\sigma \sqrt{2\pi }}e^{-{\left(\ln x-\mu \right)}^{2}/\left(2\sigma \right)},$$

where μ and σ correspond to the mean and the standard deviation of the normal distribution of lnx. We will vary the value of $x_{i}$ by again introducing a scaling factor that can be changed such that $x_{i}=zx_{0,i}$ and $\theta_{i}=-\ln\left(zx_{0,i}\right)$, where we sample $x_{0,i}$*once* from the log-normal distribution for a variety of values for μ and σ.

The log-normal distribution allows us to inspect the transition in the relationship between the overlap and the number of links from the quadratic lower limit to the linear upper limit by varying the value of σ. Indeed, when 0<σ≪1, the normal distribution of $\ln x_{0,i}$ is sharply peaked. By decreasing the value of σ towards 0, $\ln x_{0,i}$ (and, therefore, $x_{0,i}$ as well) shall approach a delta distribution. This is the distribution that led us to the quadratic lower limit for the relationship between the overlap and the number of links in the network. Conversely, when σ≫1, the log-normal distribution approaches a distribution with a power law tail with γ=1. This distribution led us to the linear upper limit between the overlap and the number of links in the network (when *J* was sufficiently large). By increasing the value of σ from 0 to a sufficiently large value (e.g., σ=10), we can therefore increase the heterogeneity of the network from a completely homogeneous network achieving the quadratic lower limit to an extremely heterogeneous network close to the linear upper limit relationship in the simulation data.

**Figure 8:**
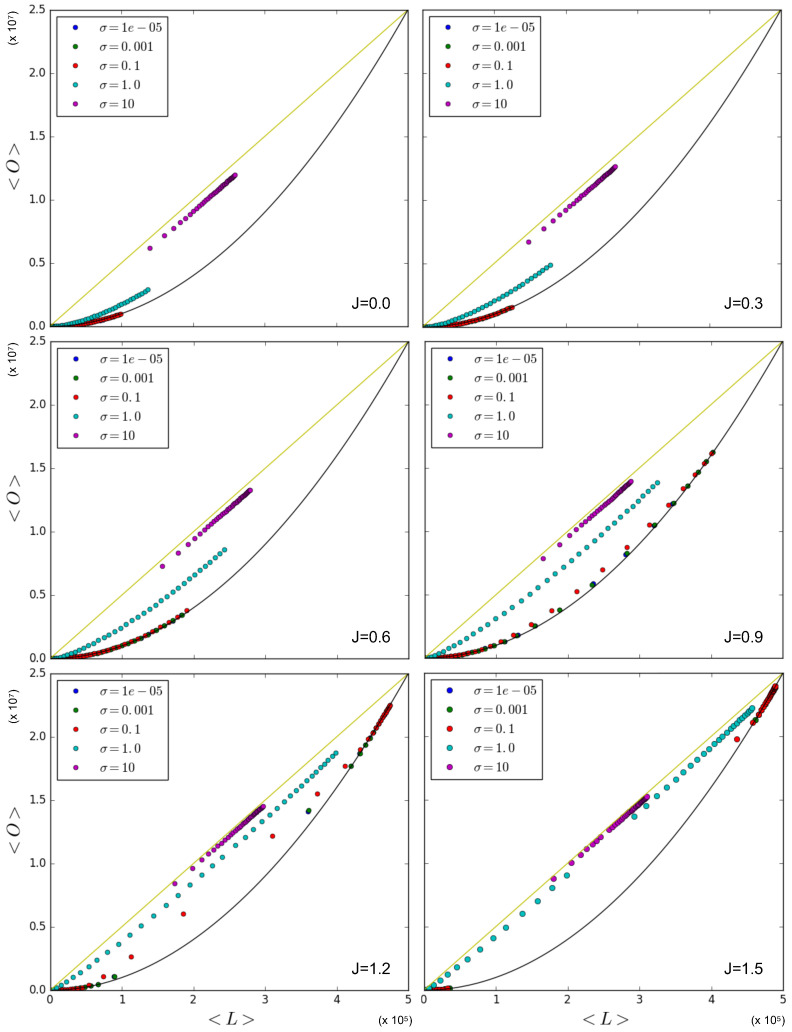
Relationship between the expected inter-layer overlap 〈O〉 and the total number of links 〈L〉 in heterogeneous multiplexes with N=100, M=100, and $x_{0,i}$ sampled from a log-normal distribution with different values for σ. The colored points correspond to simulations obtained via the Metropolis–Hastings algorithm for J∈{0.0,0.3,0.6,0.9,1.2,1.5} and z∈[0.05,2.00] in steps of Δz=0.05. The straight line corresponds to the upper limit 〈O〉=M〈L〉/2 calculated in Equation (67). The solid curve corresponds to the quadratic trend $\langle O\rangle ={\langle L\rangle }^{2}/N^{2}$ (achieved by homogeneous multiplexes with constant $x_{i}$), which here marks a lower bound achieved when $\sigma \to 0^{+}$. For increasing values of *J* (genuine coupling) and σ (spurious coupling), the system moves closer to the upper bound. For J>1, we see that, starting from the multiplexes with smaller values of σ, the points are concentrating towards high-density and low-density (symmetry-broken) regimes, drifting away from the intermediate values, like in the homogeneous and power law cases. To realize this separation for larger values of σ, a larger value of *J* is required.

Figure 8 shows the relationship between the average overlap and the number of links in the network with simulation data that were obtained by using the Metropolis–Hastings algorithm for a variety of values for *J* and σ. Again, the linear upper limit is illustrated as a straight line and the quadratic lower limit as a solid curve. The figure confirms that in the case where J=0, the data points that correspond to $x_{0,i}$ being sampled from a log-normal distribution with a relatively low value for σ are either on or close to the quadratic lower limit curve. On the other hand, the case where σ=10 results in data points where the overlap in the network for a given number of links is almost maximal, and therefore approaches the linear upper limit. This first set of results confirms the strong role of node heterogeneity in determining increased correlations between the degrees of the same node across different layers, which, in turn, increase the inter-layer overlap even without any explicit coupling (J=0), and hence, in a ‘spurious’ manner. On the other hand, when we increase the value of *J*, the data points corresponding to relatively low values of σ (e.g., $\sigma =10^{-5}$ and $\sigma =10^{-3}$) stay on or close to the quadratic lower limit, a finding similar to the results in Section 5.1.1, showing that the symmetry-broken values realized by different pairs of nodes, when averaged across the network, restore the ensemble average because the node pairs are all independent and (almost) identically distributed. Remarkably this means that, in a certain sense, node homogeneity ‘suppresses’ the effects of the true inter-layer coupling (J>0) on the realized overlap. For the intermediate value σ=1.0, the data are distributed close to the quadratic lower limit curve only for low values of *J*, while increasing the value of *J* leads to a more linear trend, eventually approaching the linear upper limit. In this case, the coupling is effective in producing a higher realized overlap. In the case where σ=10, the linear trend is instead achieved already for J=0.0 (although the points are aligned below it); hence, increasing the value of *J* barely influences the value of the overlap for a given number of links.

Therefore the effect of increasing *J* in networks with a moderate heterogeneity is a transition from multiplex configurations with densities of all levels towards multiplex configurations with either low or high density, which is a result of the phase transition. It also shows that a very high level of heterogeneity leads to an overlap in the network that is already close to maximal for a given number of links, irrespective of the phase transition and the value of *J*. However, in the case where we have an intermediate level of heterogeneity (σ=1.0), we observe that the effect of the coupling can be relatively strong, and we can therefore construct networks with a combination of the overlap and number of links falling between the extreme linear upper limit and the quadratic lower limit in a controlled, systematic manner. Note that Figure 8 also shows that, as *J* increases above 1, the (symmetry-broken) realized data start to ‘drift away’ from the intermediate densities, in a way similar to what we observed in Figure 5, but in a more pronounced manner. This is due to the fact that, as *J* increases, a larger number of multilinks shall be either in the low-density or high-density phase.

**Figure 9:**
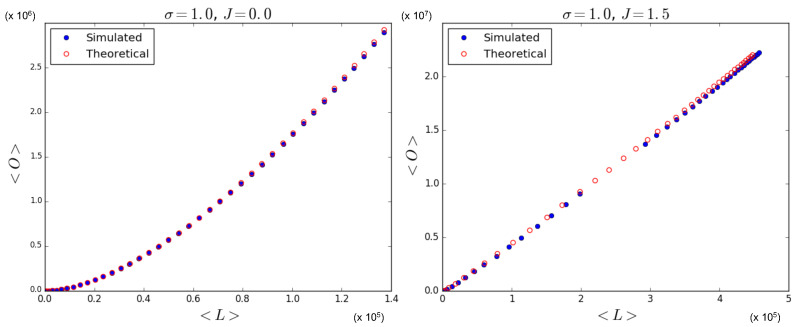
Relationship between the expected inter-layer overlap 〈O〉 and the total number of links 〈L〉 in heterogeneous multiplexes with N=100, M=100, and $x_{0,i}$ sampled from a log-normal distribution with σ=1. The blue points correspond to simulations obtained via the Metropolis–Hastings algorithm for z∈[0.05,2.00] in steps of Δz=0.05 with J=0 (**left** panel) and J=1.5 (**right** panel). The red open circles are the theoretically predicted values corresponding to the same parameters used in the simulations.

Again, in Figure 9 (which is the counterpart of Figure 7), we ‘zoom in’, and, using Equations (53), (55), and (56), we show the theoretically predicted values of 〈O〉 and 〈L〉 and compare them to the simulation data, where $x_{0,i}$ is sampled from a log-normal distribution with σ=1, for J=0 and J=1.5. The results for $\sigma \in \{10^{-5},10^{-3},10^{-1},10^{1}\}$ are not shown here since relatively low and high values for σ lead to results similar to those we have shown in Section 5.1.1 and Section 5.1.2, respectively. Figure 9 confirms that the theoretical predictions are in good agreement with the simulation data, apart from the expected ‘drifting away’ of symmetry-broken values from the corresponding ensemble average.

## 6. Analysis of the World Trade Multiplex

In this section, we finally consider an application of the model to a real-world economic network. Since our models lead to multiplex networks with independent pairs of nodes (i.e., independent multilinks) even when links are correlated across layers, it is important that the real-world network is consistent with this assumption. For instance, networks constructed from time series data [3,4,5] are not viable, because the known (and strong) correlations between the time series corresponding to different vertices generate dependencies between pairs of nodes (and higher-order patterns) through the triangular inequality [6,7]. For this reason, we select the World Trade Multiplex as an ideal case study for the present analysis, because each separate layer of that network has been successfully modeled in the past via maximum entropy models of networks with given degrees [31,32,33]. At the same time, it has been shown that certain structural properties of commodity-specific layers are very similar across the different layers of the multiplex [30], and that this similarity (in particular, the correlation among the degrees of the same node in different layers) generates a large spurious component of the inter-layer overlap [28,29], which is not necessarily due to a genuine coupling. In this sense, our analysis here will add a natural novel aspect to the modeling of the network, namely, the explicit comparison with a model with nontrivial coupling among layers, which has not been considered so far. We use the UN-COMTRADE dataset that represents the multiplex network of international trade (https://comtradeplus.un.org, accessed on 2 September 2019). The different layers of this multiplex network represent different commodities. The vertices in this network represent different countries, and a link exists between two countries in a given layer if there is trade between them in that commodity. The data include N=206 countries and M=96 commodities. Some examples of traded commodities are meat, fish, dairy products, coffee, and tobacco [30,33].

**Figure 10:**
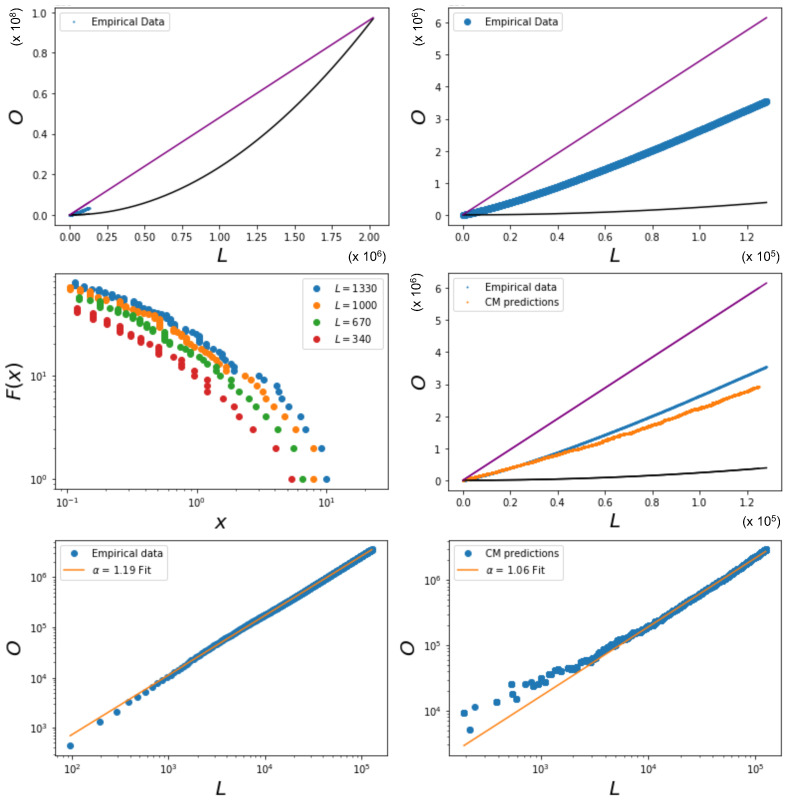
Comparison of the empirical World Trade Multiplex (WTM) with the zero-coupling ($J^{*}=0$) benchmark provided by the Average Configuration Model (ACM). The WTM consists of N=206 nodes, each representing a country, and M=96 layers, each representing a commodity group. The filtered data were obtained by retaining the same number $L^{0}$ of strongest links in each layer (hence $L=ML^{0}$ links in the entire multiplex), and varying $L^{0}$. **Top left**: relationship between the expected inter-layer overlap 〈O〉 and the total number of links 〈L〉 in the WTM (blue), compared with the upper limit 〈O〉=M〈L〉/2 calculated in Equation (67) (purple straight line) and the quadratic trend $\langle O\rangle ={\langle L\rangle }^{2}/N^{2}$ achieved by homogeneous multiplexes (black solid curve). **Top right**: zoomed-in version of the top left panel, showing that the empirical data follow an intermediate scaling between the two extremes. **Center left**: cumulative distributions reporting the number F(x) of nodes with hidden variable larger than *x* in the ACM, obtained for different values of $L^{0}$ (see legend). **Center right**: same as the top right panel with the addition of the relationship produced by the ACM benchmark, showing that the empirical WTM (blue) has a higher overlap than the corresponding null model having zero inter-layer coupling but the same degree heterogeneity (orange). **Bottom left**: log–log plot of the relationship between the overlap and the number of links in the empirical WTM, along with a power law fit of the form $O=AL^{\alpha }$, where the fitted exponent is α=1.19. **Bottom right**: log–log plot of the same relationship in the ACM benchmark with no coupling, along with a power law fit of the form $O=AL^{\alpha }$, where the fitted exponent is α=1.06.

Using the international trade data, we wish to identify a possibly nontrivial overlap by creating (L,O) plots similar to the ones depicted in Figure 6 and Figure 7 or Figure 8. We therefore repeatedly filter the network such that each layer α has the same number of links $L^{\alpha }\equiv L^{0}$ (where α=1,…,M), and calculate the corresponding overlap *O* for the specified value of $L^{0}$ (note that this means that the total number of links in the entire multiplex is $L=ML^{0}$). The criterion we follow is choosing the $L^{0}$ strongest (highest weight) links in every layer to obtain data with comparable degrees across layers, as in our models. Note that, by using this filtering method, the highest possible density we can achieve is limited by the density of the sparsest layer in the unfiltered network. The results are shown in Figure 10, which indicates that the overlap for a given number of links appears to be around halfway between the quadratic lower limit curve and the linear upper limit curve. This suggests that the degree of heterogeneity of the network is intermediate, similar to that realized by log-normally distributed fitness values, as in our example considered above.

As anticipated, we are currently unable to solve the maximum likelihood equations in order to obtain the joint values of all the Lagrange multipliers in the full OACM model with J≠0. However, after filtering the original empirical network such that every layer has $L^{0}$ links, we can use the values of the hidden variables $x_{i}^{*}$ for the null model corresponding to the absence of inter-layer coupling, i.e., to $J^{*}=0$. As we have shown in Equations (57), this assumption reduces our model to the ACM discussed in Section 2.5. The maximum likelihood equations in this case are much easier to solve, and can be found using one of the numerical algorithms available at https://meh.imtlucca.it (accessed on 1 May 2023). This procedure is repeated for a range of values for $L^{0}$. The cumulative distribution of the hidden variables $x_{i}^{*}$ are plotted in Figure 10 for various values of $L^{0}$. The figure qualitatively shows that the shape of the cumulative distribution of *x* is fat-tailed and indeed similar to the one for a log-normal distribution. Moreover, it does not vary with $L^{0}$, apart from an overall change of scale.

The null model with $J^{*}=0$, when compared to the data for the same choice of $L_{0}$, allows us to detect the presence of nontrivial coupling among the layers, when present. Indeed, from Figure 10, we see that the filtered networks have a relatively high overlap, the data points being distributed along a similar trend as the one corresponding to a nonzero *J* in our previous heterogeneous examples. By using the values of the hidden variables for the model with $J^{*}=0$, we can calculate the corresponding expected number of links and the expected overlap under the null hypothesis of no coupling between the layers, but the same average degree sequence in the real network. The results are shown in Figure 10, alongside the curve corresponding to the empirical data. We see that the assumption J=0 leads to an insufficiently overlapping multiplex, demonstrating the necessity of a model that introduces dependencies between the layers of a network. The difference between the two curves can be quantified by fitting both to the curve

(76) $$O=AL^{\alpha }\;$$

where *A* is a proportionality factor and α is an exponent (not to be confused with the label of a layer of the multiplex). For the empirical data, we find a steeper increase characterized by an exponent $\alpha_{\mathrm{empirical}}=1.19$, while for the predictions from the ACM, we find $\alpha_{\mathrm{CM}}=1.06$ (see Figure 10). The difference between the two values implies that the difference between the realized and expected overlap increases as *L* increases, confirming that the observed overlap in the WTM is not only the spurious result of the correlated heterogeneity of the degrees of countries, but reflects genuine ($J^{*}>0$) inter-layer dependencies.

We conclude with a discussion about the entropy in the heterogeneous case, analogous to the one we made in Section 5.1.1 in the homogeneous case. Here we note that, given a multiplex $\vec{G}*$ of interest, the entropy $S(\vec{\theta }*,J^{*})$ of the data, given the OACM model, is the one given by Equation (58), which in the heterogeneous case cannot be, in general, reduced to a simpler formula. However, if we define the minimum and maximum values of the hidden variables as

(77) $$\theta_{\min}^{*}\equiv \min_{i=1,N}\left\{\theta_{i}^{*}\right\},\;\theta_{\max}^{*}\equiv \max_{i=1,N}\left\{\theta_{i}^{*}\right\},\;$$

respectively, we can bound the entropy as follows:

(78) $$S_{\min}\left(\vec{\theta }*,J^{*}\right)\leq S\left(\vec{\theta }*,J^{*}\right)\leq S_{\max}\left(\vec{\theta }*,J^{*}\right)\;$$

where we have defined

(79) $$\begin{array}{ccc} S_{\min}\left(\vec{\theta }*,J^{*}\right) & \equiv & 2\theta_{\min}^{*}L^{*}-\frac{4J^{*}}{M}O^{*}+\sum_{i<j}\ln z_{ij}\left(\theta_{i}^{*}+\theta_{j}^{*},J^{*}\right), \end{array}$$

(80) $$\begin{array}{ccc} S_{\max}\left(\vec{\theta }*,J^{*}\right) & \equiv & 2\theta_{\max}^{*}L^{*}-\frac{4J^{*}}{M}O^{*}+\sum_{i<j}\ln z_{ij}\left(\theta_{i}^{*}+\theta_{j}^{*},J^{*}\right).\; \end{array}$$

The bounds in Equation (78) are alternative to the general ones in Equation (63), and arguably more useful to characterize how the entropy is effectively constrained by, once again, the relationship between $L^{*}$ and $O^{*}$. The latter, unlike the homogeneous case, is not necessarily quadratic, and can follow the diverse trends we have shown in Figure 6, Figure 8 and Figure 10. In particular, the power law relationship captured by Equation (76) for the empirical WTM provides a convenient way of bounding $S(\vec{\theta }*,J^{*})$ via Equations (78)–(80).

## 7. Conclusions

In this paper we have introduced a maximum entropy model, or ERGM, of multiplex networks with given degrees and inter-layer overlap. The model allowed us to separately control the effects of the correlations between node degrees across different layers (which lead to a spurious overlap) and that of a genuine inter-layer coupling. The nature of the enforced constraints is such that different pairs of nodes are statistically independent, even if the parameters governing them are correlated via those of the nodes they share.

For each pair of nodes, the model can be mapped exactly to a mean-field Ising model featuring a magnetization-like phase transition, which includes the possibility of (spontaneous) symmetry breaking. Given the difficulty of solving the maximum likelihood equations to obtain the values of the Lagrange multipliers corresponding to a particular real network, we first treated the Lagrange multipliers as free parameters in order to explore and analyze the properties of multiplex systems as a function of these parameters using numerical methods. Additionally, the numerical results were compared to our analytical results in order to test the validity of the latter. We have shown that the analytical equations are highly accurate. The combined result, at the level of the entire multiplex, of the properties of all node pairs is nontrivial and crucially depends on the values of the node-specific parameters, which ultimately depend on the enforced degrees.

In the fully homogeneous case, the phase transition occurs at the same critical point for all node pairs simultaneously, because the parameters are identical for all nodes. However, the independence of different node pairs implies that, even in the magnetized phase, the realized values of the inter-layer overlap and total number of links coincide with the ensemble average. This happens because different node pairs realize all the possible symmetry-broken values independently, so that an average of the realized values for a large number of independent node pairs asymptotically equals the ensemble average. The value of *J* has little effect on the relationship connecting the overlap to the number of links, which remains similar to what we observed for the case J=0, showing that node homogeneity suppresses the effects of a genuine inter-layer coupling.

In the heterogeneous case, the phenomenology is very different since, despite the fact that node pairs are still independent, they are now governed by different parameters, and the ensemble average for a given pair can no longer be realized as an average of the realized values of pairs with the same parameters. This implies that the observed overlap and number of links will depend on the realized symmetry-broken values, whose typical value does not coincide in general with the ensemble average, and is determined by the node-specific parameters (hence, ultimately by the degrees). Moreover different pairs of nodes are, in general, found in different phases, so the multiplex displays, as a function of the parameters, a hierarchy of phase transitions. We have found that increasing the value of the coupling parameter *J* generally increases the (genuine) overlap for a given number of links, if there is enough node heterogeneity. However, we have also shown that increasing the heterogeneity of the network increases the (spurious) overlap for a given number of links as well. This is a consequence of the presence of large hubs that appear in a correlated manner across layers, due to the increased heterogeneity of the network. Additionally, every multilink that is connected to these hubs has a relatively low critical threshold for the coupling parameter *J*. Therefore, these multilinks have a higher probability to be in the high density phase, which leads to a higher overlap as well, which corresponds to increasing the amount of genuine correlation. In general, the overlap for a given number of links can be increased by increasing either the heterogeneity of the network or the value of the coupling parameter, with a subtle interplay between the two. In principle, this can be used in order to create multiplexes with a specific degree of overlap for a given of number of links, provided their combination is within the theoretical limits discussed in Section 5.

Finally, by using a dataset that represents the empirical multiplex network of international trade in several commodity-specific layers, we have used the model to disentangle the spurious overlap arising from the documented strong correlation of node degrees across layers [28,29] from the genuine overlap arising from actual inter-layer coupling. We have found that the assumption that there is no coupling between the layers (J=0), which reduces our model to the ACM, results in a multiplex with insufficient inter-layer overlap. This means that the empirical overlap is not merely the spurious result of the correlated heterogeneity of the network, but requires a true nonzero coupling between layers.

Our results demonstrate the subtleties of the interplay between node heterogeneity and inter-layer dependencies in multiplex networks, highlighting the need for null models that can control these factors separately. In this paper, we have introduced perhaps the simplest, although already very rich, model of this type. Our model can be seen as a minimal one, to be further generalized in the future.

## Data Availability

For the empirical analysis of the World Trade Multiplex, the publicly available UN-COMTRADE dataset was analyzed in this study. The data are available at http://comtrade.un.org/ (accessed on 2 September 2019). The codes used for the numerical calculation of the parameters maximizing the likelihood are available at https://meh.imtlucca.it (accessed on 1 May 2023).

## Appendix A. Hubbard–Stratonovich Transform

The pair Hamiltonian of our OACM in Equation (47) can be rewritten as

(A1) $$h_{ij}\left(s_{ij},B_{ij},J\right)=-B_{ij}\sum_{\alpha =1}^{M}\sigma_{ij}^{\alpha }-\frac{J}{2M}{\left(\sum_{\alpha =1}^{M}\sigma_{ij}^{\alpha }\right)}^{2}+\frac{J}{2}+v_{ij}.$$

We want to obtain an expression for the pair partition function:

(A2) $$\begin{array}{cc} z_{ij}\left(B_{ij},J\right) & =\sum_{s_{ij}\in S_{ij}}e^{-h_{ij}\left(s_{ij},B_{ij},J\right)}\\ \; & =\sum_{s_{ij}\in S_{ij}}\exp\left[\frac{J}{2M}{\left(\sum_{\alpha =1}^{M}\sigma_{ij}^{\alpha }\right)}^{2}+B_{ij}\sum_{\alpha =1}^{M}\sigma_{ij}^{\alpha }-\frac{J}{2}-v_{ij}\right]\\ \; & =e^{-J/2}e^{-v_{ij}}\sum_{s_{ij}\in S_{ij}}\exp\left[{\left(\sqrt{\frac{J}{2M}}\sum_{\alpha =1}^{M}\sigma_{ij}^{\alpha }\right)}^{2}+B_{ij}\sum_{\alpha =1}^{M}\sigma_{ij}^{\alpha }\right]. \end{array}$$

The argument of the exponent in the above expression can be linearized by using the Gaussian integral

(A3) $$e^{a^{2}}=\frac{1}{\sqrt{2\pi }}\int_{-\infty }^{\infty }d\xi_{ij}e^{-\xi_{ij}^{2}/2+\sqrt{2}a\xi_{ij}}.$$

In our case, by choosing $a=\sqrt{J/(2M)}\sum_{\alpha =1}^{M}\sigma_{ij}^{\alpha }$ the partition function factorizes with respect to the individual summations of $\sigma_{ij}^{\alpha }$:

(A4) $$\begin{array}{ccc} z_{ij}\left(B_{ij},J\right) & = & \frac{e^{-J/2-v_{ij}}}{\sqrt{2\pi }}\sum_{s_{ij}\in S_{ij}}\int_{-\infty }^{\infty }d\xi_{ij}e^{-\xi_{ij}^{2}/2}\exp\left[\sum_{\alpha =1}^{M}\sigma_{ij}^{\alpha }\left(\sqrt{\frac{J}{M}}\xi_{ij}+B_{ij}\right)\right]\\ & = & \frac{e^{-J/2-v_{ij}}}{\sqrt{2\pi }}\int_{-\infty }^{\infty }d\xi_{ij}e^{-\xi_{ij}^{2}/2}\sum_{\sigma_{ij}^{1}\in \left\{-1,1\right\}}\;\;\ldots \;\;\sum_{\sigma_{ij}^{M}\in \left\{-1,1\right\}}\prod_{\alpha =1}^{M}\exp\left[\sigma_{ij}^{\alpha }\left(\sqrt{\frac{J}{M}}\xi_{ij}+B_{ij}\right)\right]\\ & = & \frac{e^{-J/2-v_{ij}}\;2^{M}}{\sqrt{2\pi }}\int_{-\infty }^{\infty }d\xi_{ij}e^{-\xi_{ij}^{2}/2}{\left[\cosh\left(\sqrt{\frac{J}{M}}\xi_{ij}+B_{ij}\right)\right]}^{M}. \end{array}$$

Performing the change of variable $\sqrt{J/M}\xi_{ij}=Jy_{ij}$ we obtain

(A5) $$z_{ij}\left(B_{ij},J\right)=2^{M}\sqrt{\frac{JM}{2\pi }}e^{-J/2}e^{-v_{ij}}\int_{-\infty }^{\infty }d\xi_{ij}{\left[\Phi_{J,B_{ij}}\left(y_{ij}\right)\right]}^{M}$$

where

(A6) $$\Phi_{J,B_{ij}}\equiv e^{-Jy_{ij}^{2}/2}\cosh\left(Jy_{ij}+B_{ij}\right).$$

We are interested in the large *M* limit. To proceed in the calculation of $z_{ij}\left(B_{ij},J\right)$, it is useful to define the quantity

(A7) $$f_{ij}\left(B_{ij},J\right)\equiv -\lim_{M\to \infty }\frac{1}{M}\ln z_{ij}\left(B_{ij},J\right)=-\lim_{M\to \infty }\ln z_{ij}^{1/M}\left(B_{ij},J\right)$$

which is the *free energy per layer*. By inserting the result (A5) into (A7), we obtain

(A8) $$\begin{array}{ccc} f_{ij}\left(B_{ij},J\right) & = & -\ln2-\lim_{M\to \infty }\frac{1}{M}\left[\ln\left(e^{-J/2}\sqrt{\frac{JM}{2\pi }}\right)-v_{ij}+\ln\left(\int_{-\infty }^{\infty }dy_{ij}{\left[\Phi_{J,B_{ij}}\left(y\right)\right]}^{M}\right)\right]\\ & = & -\ln2+\frac{J}{2}-B_{ij}-\ln\left[\lim_{M\to \infty }{\left(\int_{-\infty }^{\infty }dy_{ij}{\left[\Phi_{J,B_{ij}}\left(y\right)\right]}^{M}\right)}^{1/M}\right]. \end{array}$$

In order to obtain a more explicit form of the function $f_{ij(B_{ij},J)}$, we use the Laplace theorem [49]. Let ϕ(y) and ψ(y) be continuous and positive functions within a range c≤y≤d, then

(A9) $$\lim_{M\to \infty }{\left[\int_{c}^{d}\psi \left(y\right){\left(\phi (y)\right)}^{M}\right]}^{1/M}=\max_{c\leq y\leq d}\phi \left(y\right).$$

For ψ(y)=1 and $\phi \left(y\right)=\Phi_{J,B_{ij}}\left(y\right)$, this results in

(A10) $$f_{ij}\left(B_{ij},J\right)=-\ln2+\frac{J}{2}-B_{ij}-\ln\left[\max_{-\infty \leq y_{ij}\leq \infty }\Phi_{J,B_{ij}}\left(y_{ij}\right)\right].$$

The derivative of $\Phi_{J,B_{ij}}\left(y_{ij}\right)$ with respect to $y_{ij}$ is zero at its maximum:

(A11) $$\frac{d\Phi_{J,B_{ij}}\left(y_{ij}\right)}{dy_{ij}}=Je^{-Jy_{ij}^{2}/2}\sinh\left(Jy_{ij}+B_{ij}\right)-Jy_{ij}e^{-Jy_{ij}^{2}/2}\cosh\left(Jy_{ij}+B_{ij}\right)=0.$$

The variable $y_{ij}$ therefore obeys the equation

(A12) $$y_{ij}=\tanh\left(Jy_{ij}+B_{ij}\right).$$

Note that this equation is identical to the one obtained for the magnetization in the Ising Model, and, depending on the values of *J* and $B_{ij}$, there are either one or three solutions that satisfy Equation (A12). The free energy $f_{ij}$ can now be written as a function of *J* and $B_{ij}$:

(A13) $$f_{ij}\left(B_{ij},J\right)=-\ln2+\frac{J}{2}-B_{ij}+\frac{J}{2}{\left(y_{ij}\right)}^{2}-\ln\left[\cosh\left(Jy_{ij}+B_{ij}\right)\right].$$

We then finally arrive at the pair partition function

(A14) $$\begin{array}{ccc} z_{ij}\left(B_{ij},J\right) & = & e^{-Mf_{ij}}\\ & = & 2^{M}e^{-v_{ij}}e^{-JM{\left(y_{ij}\right)}^{2}/2}\cosh^{M}\left(Jy_{ij}+B_{ij}\right) \end{array}$$

which, returning to the variables $\theta_{ij}$, coincides with Equation (52) in the main text, where $u_{ij}$ is the solution to Equation (53).

## Appendix B. Maximum Likelihood

To determine the parameters $\left(\vec{\theta }*,J^{*}\right)$ that maximize the log-likelihood of the OACM given in Equation (54), we first calculate the derivatives

(A15) $$\begin{array}{ccc} -\frac{\partial L(\vec{\theta },J)}{\partial \theta_{k}} & = & \sum_{i<j}\frac{\partial h_{ij}\left(m_{ij}^{*},\theta_{ij},J\right)}{\partial \theta_{k}}+\sum_{i<j}\frac{\partial \ln z_{ij}\left(\theta_{ij},J\right)}{\partial \theta_{k}},\;k=1,\ldots ,N \end{array}$$

(A16) $$\begin{array}{ccc} -\frac{\partial L(\vec{\theta },J)}{\partial J} & = & \sum_{i<j}\frac{\partial h_{ij}\left(m_{ij}^{*},\theta_{ij},J\right)}{\partial J}+\sum_{i<j}\frac{\partial \ln z_{ij}\left(\theta_{ij},J\right)}{\partial J}. \end{array}$$

We then set the derivatives with respect to $\theta_{k}$ to zero:

(A17) $$\begin{array}{cc} -{\left.\frac{\partial L(\vec{\theta },J)}{\partial \theta_{k}}\right|}_{\vec{\theta }*,J^{*}}\; & =\sum_{\alpha =1}^{M}\sum_{j\neq k}{g_{jk}^{*}}^{\alpha }-M\sum_{j\neq k}u_{jk}^{*}=0 \end{array}$$

where we utilize the fact that $g_{ij}^{\alpha }$ and $u_{ij}$ are symmetric with respect to the indices i,j, i.e.,

(A18) $$\sum_{i<j}g_{ij}^{\alpha }\delta_{i}^{k}=\sum_{j=k+1}^{N}g_{jk}^{\alpha },\;\sum_{i<j}g_{ij}^{\alpha }\delta_{j}^{k}=\sum_{j=1}^{k-1}g_{jk}^{\alpha }.$$

Similarly, we set the derivative with respect to *J* to zero:

(A19) $$\begin{array}{c} -{\left.\frac{\partial L(\vec{\theta },J)}{\partial J}\right|}_{\vec{\theta }*,J^{*}}=\sum_{i<j}\left(-\frac{4}{M}\sum_{\alpha <\beta }{g_{ij}^{*}}^{\alpha }{g_{ij}^{*}}^{\beta }+2M{\left(u_{ij}^{*}\right)}^{2}\right)=0. \end{array}$$

Taken together, the above calculations lead to the maximum likelihood Equations (55) and (56) in the main text.
